# Clinical and prognostic significance of small paroxysmal nocturnal hemoglobinuria clones in myelodysplastic syndrome and aplastic anemia

**DOI:** 10.1038/s41375-021-01190-9

**Published:** 2021-03-04

**Authors:** Bruno Fattizzo, Robin Ireland, Alan Dunlop, Deborah Yallop, Shireen Kassam, Joanna Large, Shreyans Gandhi, Petra Muus, Charles Manogaran, Katy Sanchez, Dario Consonni, Wilma Barcellini, Ghulam J. Mufti, Judith C. W. Marsh, Austin G. Kulasekararaj

**Affiliations:** 1 Department of Hematological medicine, King’s College Hospital, London, UK; 2 Hematology, Fondazione IRCCS Ca’ Granda Ospedale Maggiore Policlinico di Milano, Milan, Italy; 3 Department of Oncology and Onco-hematology, University of Milan, Milan, Italy; 4 Hematological Medicine, King’s College London, London, UK

**Keywords:** Anaemia, Myelodysplastic syndrome

## Abstract

In this large single-centre study, we report high prevalence (25%) of, small (<10%) and very small (<1%), paroxysmal nocturnal hemoglobinuria (PNH) clones by high-sensitive cytometry among 3085 patients tested. Given PNH association with bone marrow failures, we analyzed 869 myelodysplastic syndromes (MDS) and 531 aplastic anemia (AA) within the cohort. PNH clones were more frequent and larger in AA vs. MDS (*p* = 0.04). PNH clone, irrespective of size, was a good predictor of response to immunosuppressive therapy (IST) and to stem cell transplant (HSCT) (in MDS: 84% if PNH+ vs. 44.7% if PNH−, *p* = 0.01 for IST, and 71% if PNH+ vs. 56.6% if PNH− for HSCT; in AA: 78 vs. 50% for IST, *p* < 0.0001, and 97 vs. 77%, *p* = 0.01 for HSCT). PNH positivity had a favorable impact on disease progression (0.6% vs. 4.9% IPSS-progression in MDS, *p* < 0.005; and 2.1 vs. 6.9% progression to MDS in AA, *p* = 0.01), leukemic evolution (6.8 vs. 12.7%, *p* = 0.01 in MDS), and overall survival [73% (95% CI 68–77) vs. 51% (48–54), *p* < 0.0001], with a relative HR for mortality of 2.37 (95% CI 1.8–3.1; *p* < 0.0001) in PNH negative cases, both in univariate and multivariable analysis. Our data suggest systematic PNH testing in AA/MDS, as it might allow better prediction/prognostication and consequent clinical/laboratory follow-up timing.

## Introduction

The presence of paroxysmal nocturnal hemoglobinuria (PNH) clones in bone marrow failure syndromes has been demonstrated by various investigators in heterogeneous series. The rate of positivity varies greatly between 4 and 40% largely depending on the method and on the sensitivity [1–6]. Since the last 10 years, a novel cytofluorimetric technique (fluorescent aerolysin (FLAER)-based assay) has been routinely used to detect PNH clones, with a sensitivity of ≥0.01% clone size. The significance of subclinical, small PNH clones (<10%) in the context of bone marrow failure syndromes is still debatable, with some evidences for better response to immunosuppressive therapy (IST) in aplastic anemia (AA) cases [7, 8]. Moreover, although generally considered unremarkable, the recent observation of asymptomatic end-organ damage due to the undiagnosed thrombosis even in patients without overt hemolysis questions the assertion that small erythrocyte PNH clones are of subclinical value [9]. In this study, we aimed to determine the prevalence of PNH clones of any size (<1, 1–10, 10–50, >50%) by flow cytometry with FLAER in an unselected population referred to a tertiary hematology center in UK. We correlated clone size with disease category (particularly AA and myelodysplastic syndromes, MDS), clinical and laboratory features, number, type and response to therapies, and with the occurrence of thrombosis, disease progression/evolution, and survival.

## Subjects and methods

Overall, 3085 patients (*n* = 3085) with a first time test for PNH at King’s College Hospital in London from March 1998 until October 2017 were included in the analysis. Demographic and clinical phenotypes were retrospectively evaluated from August 2017 until January 2018.

Clinical history, blood counts (hemoglobin Hb, platelets Plt, absolute neutrophil counts ANC), hemolytic parameters, bone marrow features, disease-specific categories (WHO MDS categories), severity scores (Camitta criteria for AA, international prognostic scoring system IPSS and IPSS-revised for MDS), number and type of therapeutic interventions and their response rates, occurrence of complications (particularly thrombosis), disease progression/evolution, and death were collected. Blood counts were collected at the time of PNH clone testing, hence the positive impact of transfusions on baseline hemoglobin and platelets cannot be excluded in transfusion-dependent patients. Bone marrow data were available for all patients classified as MDS (either hypoplastic or not), AA, leukemia, and myeloproliferative neoplasms (MPN).

PNH testing had been performed by classical cytometry technique until 2010 and, thereafter, using high sensitivity (≥0.01%) FLAER-based assay according to 2010 International Clinical Cytometry Society (ICCS) PNH Consensus Guidelines and 2012 Practical PNH Guidelines [10, 11]. FLAER/CD33/CD15/CD45 and FLAER/CD59 panels had been used for white blood cell and red blood cell testing, respectively. Since 2015, we have used CD157 and this has been validated since 2017 in our laboratory. CD157 is used to replace CD24 and CD14 in predicate four color granulocyte and monocyte assays, respectively, and ICCS 2013 recommends single-tube assay with CD157 to monitor both granulocytes and monocytes (see also “Supplementary methods”, “Supplementary material for CD157 validation”, and “Supplementary material for flow cytometric testing”).

Patients were divided into five groups according to granulocyte clone size (0, 0.01–1, 1–10, 10–50, and >50%) and correlations with the abovementioned clinical and hematologic characteristics were performed. An analysis to evaluate the dynamic changes of the clone size and to assess the frequency of spontaneous resolution/clone enlargement was also done for positive PNH cases that had been tested at least twice (the first and the most recent one if tested multiple times). Fluctuations were defined as growing/declining of granulocyte clone size ≥5%.

Prevalence, size, and fluctuations of PNH clones were firstly evaluated on the whole pooled population tested in the study period, encompassing several hematologic and non-hematologic conditions. Analysis of clinical impact of PNH clones was subsequently performed on patients with complete information on disease-specific risk scores, therapies, and relative responses. Survival assessment was firstly done on the whole population, and then restricted to MDS and AA for multivariable analysis.

Student’s *t* test was used for continuous variables and chi-square test for categorical ones. Analysis of variance was performed by using mean, median, ranges, and standard errors. Once identified variables associated with the occurrence of complications, response to therapy, and disease progression/evolution, relapse-free survival and overall survival (OS) hazard ratios for 95% confidence intervals was calculated by cox regression models. Cumulative incidence of relapse and of evolution, as well as OS, was evaluated by Kaplan–Meier method. All analyses were performed with Stata software [12].

## Results

### Whole cohort analysis

**Fig. 1:**
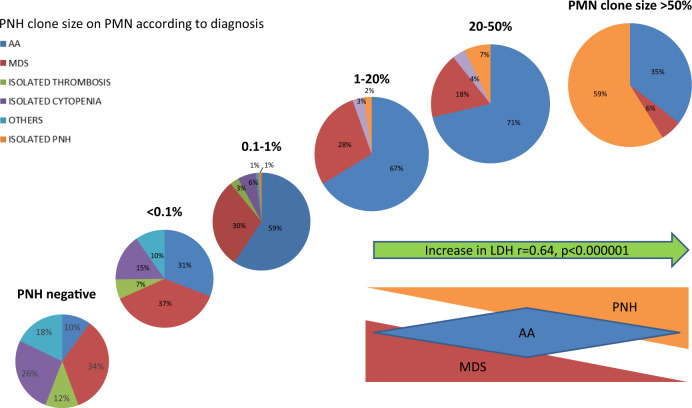
PNH clone size distribution according to the different diagnosis. MDS cases mostly presented with small (<10%) and very small clones (<1%), while classical PNH with large clones (>50%), and AA cases mainly medium clones (10–50%). LDH significantly increase along with clone size (*p* < 0.00001).

We included, in the analysis, 3085 patients with suspected underlying myeloid disorders, cytopenia, or unexplained thrombosis. They were tested from March 1998 until October 2017, all cases except 389 underwent testing with FLAER method. Median age was 53 years (0–91, IQR 29), male to female ratio was 1.08, median Hb value was 103 g/L (80–198 g/L), ANC 1.63 × 10^9^/L (range 0–92), Plt 99 × 10^9^/L (range 0–1360), and LDH levels 211 U/L (range 70–4614, normal range for LDH < 240 IU/L). PNH clone of any size greater than 0.01% on granulocytes was found in 774 cases (25%). Clinical characteristics for all patients divided according to PNH clone positivity at baseline are shown in Table 1. Patients with PNH clones were significantly younger (*p* < 0.0001), more anemic [100 g/L (40–170) vs. 105 g/L (80–198), *p* = 0.01], thrombocytopenic [72 × 10^9^/L (1–360) vs. 114 × 10^9^/L (10–980) *p* = 0.0001], and more frequently pancytopenic (*p* < 0.0001), with higher LDH levels [245 U/L (70–4614) vs. 212 (92–1520) *p* = 0.0001]. Although patients had a spectrum of different underlying diagnosis, cases who were PNH positive at baseline were more frequently treated (78% vs. 60%, *p* < 0.0001) and transfused (76% vs. 63%, *p* < 0.0001), and Eculizumab had been administered in 133 PNH+ cases (Supplementary Tables 1, 2, 3, and 5). Considering different granulocytes clone size (0, 0.01–1, 1–10, 10–50, and >50%), we observed a relationship with younger age, thrombocytopenia, and increased LDH levels (Table 2). Figure 1 shows clone size distribution according to the different diagnosis/reasons for testing. The proportion of MDS cases declined and that of classical PNH cases augmented along with clone size increase. The number of AA cases progressively increased from 0.1 to 50% granulocyte clone size, and then decreased in the >50% category. The prevalence of thrombotic episodes was comparable among PNH+ and PNH− groups, however clone size analysis, in the PNH+ cohort, showed more frequent thrombosis in the presence of larger clones (7% in cases with clone size 0.01–1% vs. 21% for clones >50%, *p* < 0.0001) (Table 2). Importantly, patients tested because of isolated thrombosis without cytopenias (7/245, 2.8%) always displayed clone size <10% and with no increase in PNH clone or hemolytic markers during follow-up.

**Table 1:** Clinical characteristics of the entire cohort at baseline and complications during follow-up.

	PNH neg	PNH pos
Number of patients, *N* (%)	2311 (75)	774 (25)
Male/female ratio	1.17	1.05
median age, years (IQR)	55 (39–67)	47 (32–64)*
Reason for testing	*N* = 2160	*N* = 744
MDS, *N* (%)	693 (32)	176 (24)*
AA, *N* (%)	204 (9)	327 (44)*
MDS/AA, *N* (%)	5 (0.2)	22 (3)*
Isolated hemolytic PNH, *N* (%)	0 (0)	97 (13)
MPN, *N* (%)	76 (4)	16 (2)
MDS/MPN, *N* (%)	92 (4)	9 (1)*
Acute leukemia, *N* (%)	209 (10)	29 (4)*
Isolated cytopenia, *N* (%)	535 (25)	51 (7)*
Thrombosis without cytopenia, *N* (%)	284 (13)	17 (2)*
Others, *N* (%)	62 (3)	0 (0)
Thrombosis occurrence, *N* (%)	370 (17)	96 (13)
Death, *N* (%)	725 (34)	141 (19)*
Lost to follow-up, *N* (%)	146 (7)	75 (10)
Median follow-up, years (range)	2 (0.5–15)	3.4 (3–16)
Hematologic parameters	*N* = 1027^a^	*N* = 744
Hb < 100 g/L, *N* (%)	409 (40)	351 (47)**
PLT < 100 × 10^9^/L, *N* (%)	463 (45)	423 (57)*
ANC < 1.5 × 10^9^/L, *N* (%)	478 (46.5)	342 (46)
Pancytopenia, *N* (%)	160 (16)	183 (25)*
Median LDH U/L (range)	212 (92–1520)	245 (70–4614)*

Values are shown for all patients tested (negative, *N* = 2311, and positive, *N* = 774).

*MDS* myelodysplastic syndromes, *AA* aplastic anemia, *MDS/AA* hypoplastic MDS, *MPN* myeloproliferative neoplasms, *Hb* hemoglobin, *PLT* platelets, *ANC* absolute neutrophil counts.

*p < 0.0005, **p = 0.01.

^a^Pre-treatment hematologic parameters at diagnosis were available only for about half PNH negative patients and were included in the analysis.

**Table 2:** Clinical characteristics of patients divided according to PNH clone size on granulocytes at baseline.

Clone size	Neg *N* = 1740	0.01–1% *N* = 215	1–10% *N* = 108	10–50% *N* = 59	>50% *N* = 128
Males, *N* (%)	945 (54)	114 (53)	60 (56)	30 (51)	56 (44)
Females, *N* (%)	795 (46)	101 (47)	48 (44)	29 (49)	72 (56)
Median age, years (IQR)	55 (39–66)	49 (34–63)	48 (30–65)	48 (28–60)	44 (31–85)
MDS, *N* (%)	616 (35)	71 (33)	32 (30)	13 (22)	7 (6)*
AA, *N* (%)	175 (10)	98 (46)	72 (67)	40 (68)	47 (37)
MDS/AA, *N* (%)	92 (5)	8 (4)	2 (2)	0 (0)	0 (0)
Acute leukemia, *N* (%)	157 (9)	11 (5)*	0 (0)	1 (2)	0 (0)
Hemolytic PNH, *N* (%)	0 (0)	1 (0.5)^a^	1 (0.5)^a^	3 (5)	74 (58)*
MPN, *N* (%)	0 (0)	8 (4)	0 (0)	0 (0)	0 (0)
MDS/MPN, *N* (%)	0 (0)	4 (2)	0 (0)	0 (0)	0 (0)
Isolated cytopenia, *N* (%)	433 (25)	19 (9)	1 (1)	1 (2)	0 (0)
Isolated thrombosis, *N* (%)	245 (14)	6 (3)*	1 (1)	0 (0)	0 (0)
Others, *N* (%)	22 (2)	0 (0)	0 (0)	0 (0)	0 (0)
	*N* = 1740	*N* = 215	*N* = 108	*N* = 59	*N* = 128
Treated, *N* (%)	425 (56)	130 (68)	81 (78)	46 (78)	114 (89)*
Thrombosis, *N* (%)	315 (18)	16 (7)	6 (6)	3 (5)	27 (21)*
Death, *N* (%)	536 (31)	33 (15)	12 (11)	12 (20)	9(7)*
	*N* = 771	*N* = 215	*N* = 108	*N* = 59	*N* = 128
Hb < 100 g/L, *N* (%)	299 (39)	102 (47)	58 (54)	30 (51)	67 (52)
PLT < 10 × 10^9^/L, *N* (%)	351 (45)	133 (62)	88 (82)	45 (76)*	43 (34)
ANC < 1.5 × 10^9^/L, *N* (%)	220 (29)	65 (30)	57 (53)	29 (49)	17 (13)
Median LDH U/L (range)	197 (73–1520)	222 (83–1200)	198 (108–1158)	266 (70–984)	880 (97–4403)*

Values are shown only for patients with evaluable granulocyte PNH clone size at baseline by FLAER and with available clinical and laboratory data (Negative, *N* = 1740, positive, *N* = 510).

*MDS* myelodysplastic syndromes, *AA* aplastic anemia, *MDS/AA* hypoplastic MDS, *MPN* myeloproliferative neoplasms, *Hb* hemoglobin, *PLT* platelets, *ANC* absolute neutrophil counts.

*p < 0.0005.

^a^Two patients showed small and very small clones at baseline and then developed classic hemolytic PNH, with 15% and 20% clone size, respectively.

Considering clone size dynamics, 230 cases underwent clone testing at least twice, and mean clone size progressively increased on all tested cell populations (on average from 9% to 38%, from 25% to 63%, and from 22% to 62% on erythrocytes, neutrophils, and monocytes, respectively) in 74 patients. We observed that MDS cases more often showed stable clones, and AA cases had the same proportion of increasing, declining, and stable clones. Patients with classic PNH displayed fluctuations (*p* < 0.0001), and those on eculizumab demonstrated enlarging PNH granulocyte clones (*p* = 0.02, Supplementary Table 1). Interestingly, PNH clone disappeared in 17 patients (7.4%), all with smaller PNH clone at baseline [median 0.1% (0.1–52.6) vs. 45.9% (0.2–100), *p* < 0.0001] and lower LDH levels [median 183 U/L (99–737) vs. 275 U/L (97–3413), *p* = 0.01] compared to those with larger clone and higher LDH.

### Subgroup analysis: MDS, AA, and other myeloid malignancies

#### MDS

**Fig. 2:**
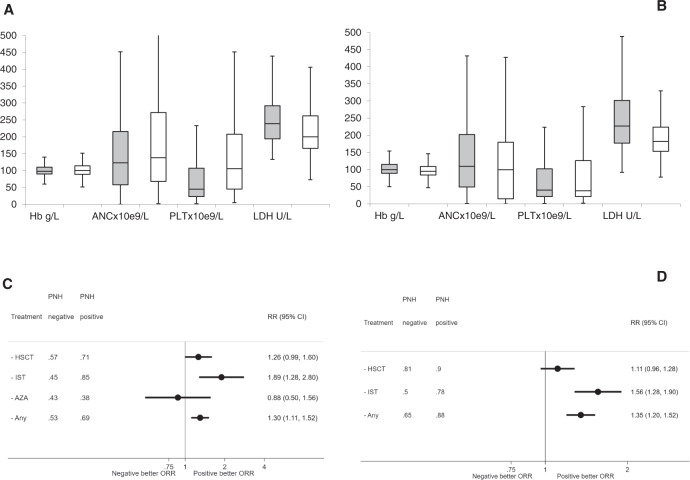
Hematologic parameters and response rates in myelodysplastic syndromes (MDS) and aplastic anemia (AA) cases according to PNH positivity. **A** Median blood counts and LDH levels +SD in PNH+ (gray bars) and PNH− (white bars) MDS cases; *p* = 0.04 for Hb; *p* < 0.0001 for PLT and LDH. **B** Median blood counts and LDH levels ± SD in PNH+ (gray bars) and PNH− (white bars) AA cases; *p* < 0.0001 for PLT and LDH. Blood counts were collected at the time of PNH clone testing, hence the positive impact of transfusions on baseline hemoglobin and platelets cannot be excluded in transfusion-dependent patients. **C** Forest plot of relative risk for response to treatments in PNH+ and PNH− MDS cases. **D** Forest plot of relative risk for response to treatments in PNH+ and PNH− AA cases. Good outcome after HSCT was considered as 6 month persistent disease remission. Hb hemoglobin, ANC absolute neutrophil counts, PLT platelets, LDH lactate dehydrogenase, HSCT hematopoietic stem cell transplant, IST immunsuppressive therapy, AZA azacytidine, ORR overall response rate.

Considering MDS separately, 176 cases (20.3%) showed the presence of PNH clone (Supplementary Figs. 1–4). As shown in Supplementary Table 2, PNH+ MDS cases were significantly more often hypoplastic, mainly displayed IPSS low/int-1 score, deeper cytopenias (especially for Hb and PLT), and higher LDH levels (Fig. 2A). Median blast count and cytogenetic aberrations (including -7 and complex karyotype) were equally distributed in the two groups, and so was revised IPSS-revised risk (Supplementary Table 2). Considering therapies, PNH positive cases were more frequently treated with IST (i.e., cyclosporine and ATG, *p* = 0.0001), and less frequently with chemotherapy and azacytidine (*p* < 0.0001 and *p* = 0.002, respectively); eltrombopag, lenalidomide, and bone marrow transplant were equally used in the two groups, and seven PNH + MDS cases received eculizumab due to thrombosis (2) or hemolysis (5). PNH+ MDS showed significantly higher response rates to IST (ATG and CyA, 84% vs. 44.7%, *p* = 0.01) and to HSCT (71% vs. 56.6%, *p* = 0.09) compared to PNH−, but not to azacytidine (Fig. 2C). The cumulative probability of response to any treatment significantly improved along with clone size increase (from 52 to 100%, *p* = 0.03). Regarding HSCT, PNH+ patients were younger (12% aged >60 years vs. 28% in PNH negative group, *p* = 0.001), and more frequently displayed low/int-1 IPSS risk at sampling (70% vs. 54%, not significant).

Regarding particular subgroups, a sub-analysis of MDS with excess of blasts (EB1/2) revealed a prevalence of 15% PNH clones, with a median clone size of 0.2% (0.01–20.7) on granulocytes (Supplementary Table 2.1, Supplementary Fig. 5). PNH positivity did not impact on treatment choice or response in this category. The subgroup of patients with hypoplastic MDS (Supplementary Table 2.2) showed high prevalence of small PNH clones (43%, with a median clone size of 0.4% on granulocytes) that were related to lower Hb and PLT levels and higher LDH. Treatment choice and responses according to PNH positivity showed the same trends as those of the whole MDS population. Finally, PNH + MDS had a numerically higher incidence of thrombotic events during follow-up (from 5% in PNH− to 9% in PNH+) but did not reach statistical significance.

#### AA

Focusing on AA, 327 patients (61.6%) displayed a PNH clone (Supplementary Fig. 6). Overall, 41% of cases had severe or very severe AA (Supplementary Table 3). PNH+ AA showed deeper thrombocytopenia, higher reticulocyte counts and LDH values (Fig. 2B). Thrombotic events were equally observed in PNH+ and PNH− patients. As observed for PNH + MDS, PNH + AA showed higher response rates to standard treatments compared to PNH− (97 vs. 77% for HSCT, *p* = 0.01; 78 vs. 50% for IST, *p* < 0.0001, and 88% vs. 65% considering any treatment, *p* < 0.0001; Fig. 2D).

By comparing MDS and AA, the frequency of PNH positivity and clone size were lower in the former (*p* = 0.04). Moreover, also considering PNH + MDS/AA separately, the analysis of clone size confirmed that the depth of cytopenias, LDH levels and thrombotic episodes increased in patients with larger clone size.

#### Other myeloid malignancies

Finally, among patients with myeloid malignancies other than MDS, we found a prevalence of PNH positivity in 17%, 9%, and 12% of patients with MPN, MPN/MDS, and acute leukemia (AL), respectively (Supplementary Figs. 7–11). All patients had been tested at diagnosis as part of the initial workup of anemia with LDH elevation (Supplementary Table 2.1). All had a clone size <1%, except for one MPN and two AL patients with a granulocyte clone between 1 and 10% by FLAER (Supplementary Table 2.1). Of note, three patients with MPN (one myelofibrosis and two chronic myeloid leukemia, CML) and five with AL were tested at least twice by FLAER (Supplementary Table 1) and granulocyte clones disappeared in one, remained stable in seven, whilst no patients had increased clone size. Importantly, in four patients with AL tested before and after chemotherapy PNH clones remained stable, whilst PNH clone disappeared in another AL subject after HSCT. Finally, in the two patients with CML re-tested after the start of tyrosine kinase inhibitor treatment, PNH clone remained stable.

### Disease outcome and survival analysis

**Fig. 3:**
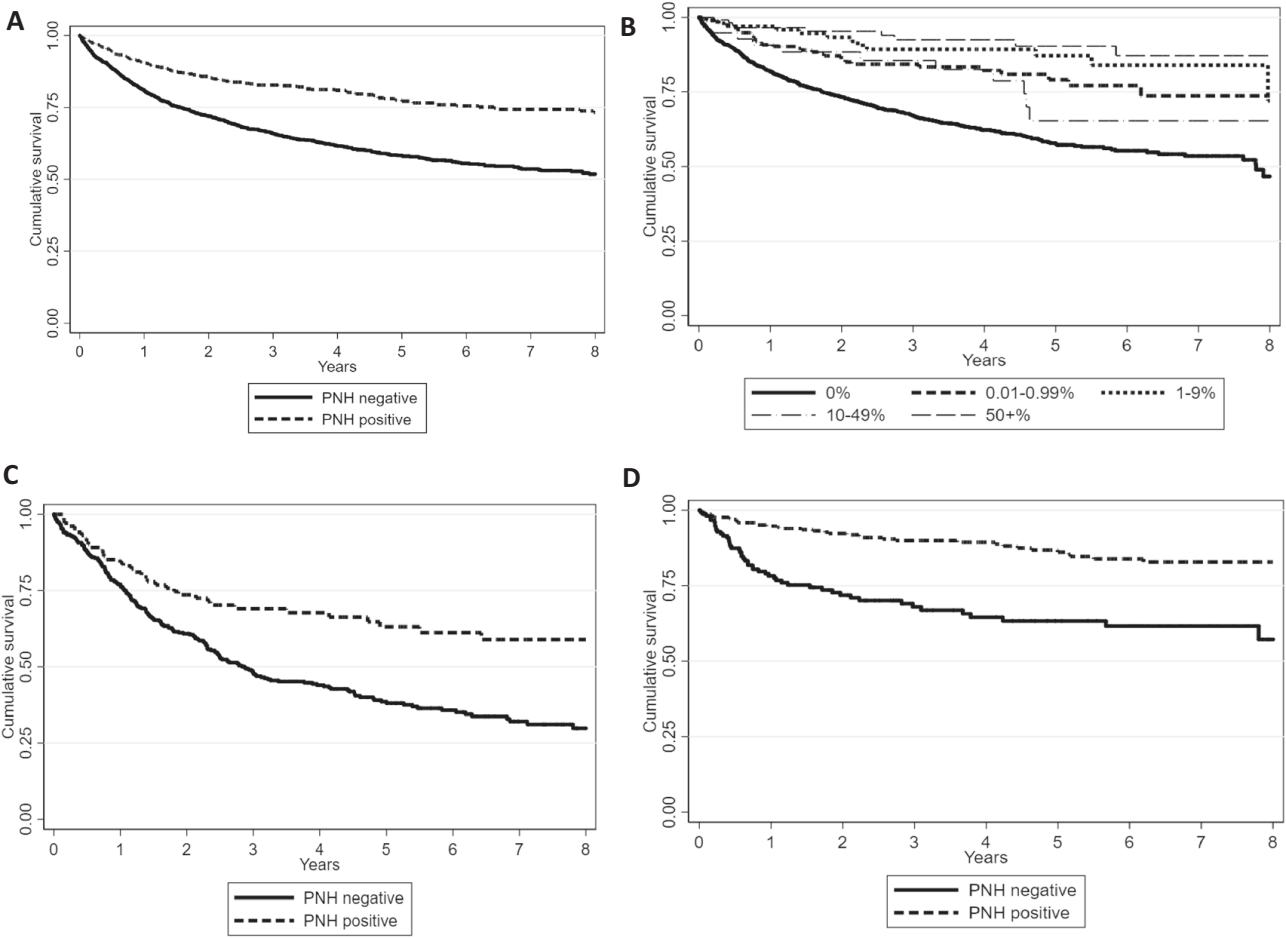
Overall survival (OS) at 8 years in PNH positive and PNH negative patients. **A** OS according to PNH positivity. **B** OS according to clone size 0.01–1, 1–10, 10–50, and >50%. **C** 8 years OS in PNH+ and PNH− MDS cases [mean OS 11.9 + 0.7 years (10.5–13.3) in PNH+ vs. 7.3 + 0.3 (6.6–7.9) in PNH−, *p* < 0.0001]. **D** 8 years OS in PNH+ and PNH− AA cases [mean OS 15.8 + 0.43 years (14.9–16.7) in PNH+ vs. 6.5 + 0.35 (5.8–7.21) in PNH−, *p* < 0.0001].

PNH+ MDS showed lower rate of IPSS-risk progression and AML evolution compared to PNH− cases; similarly, PNH+ AA less frequently evolved to MDS or AML compared to PNH− cases (Supplementary Tables 2 and 3). A total of 858 cases died, more frequently in the PNH negative group (35 vs. 19%, *p* < 0.0001). In univariate analysis, PNH− cases displayed significantly worse OS at 8 years compared to PNH+ ones [51% (95% CI 48–54) vs. 73% (68–77), *p* < 0.0001], with a relative HR of 2.37 (95% CI 1.8–3.1; *p* < 0.0001). Other factors associated to worse OS were: older age [58% (95% CI, 53–64) for >40 years of age vs. 77% (65–86) in younger patients], male gender [49% (45–52) vs. 68% (64–71) for females], transfusion dependency [54% (49–58) vs. 81% (75–86)], and presence of anemia, thrombocytopenia, and neutropenia. Multivariable analysis (Supplementary Table 4) confirmed the negative impact of PNH negativity on OS, with HR of 2.28 (1.18–4.4; *p* = 0.01), together with age >40 years, and male gender (*p* = 0.03 and *p* = 0.01, respectively), whilst the diagnosis of classic PNH was protective (*p* = 0.04). Focusing on clone size, patients with any PNH positivity (0.01–1, 1–10, 10–50, >50% clone size) showed a lower frequency of fatalities compared with negative ones (Fig. 3A, B). Notably, this was also true for PNH+ patients with 0.01–1% clone size [HR 0.43 (95% CI 0.3–0.6), (*p* < 0.0001)]. Finally, each 10% increase in clone size resulted in a 1% decrease of cumulative incidence of death. The favorable impact of PNH positivity on OS was confirmed in a separate analysis of MDS and AA cases (Fig. 3B, C). PNH positivity retained a favorable impact on OS even considering MDS-EB1/2 subcategory (Supplementary Table 2.1). As expected, worse OS also significantly correlated with older age, male gender, transfusion dependence, MDS progression/AML evolution, higher IPSS or IPSS-R score, and non-response to therapy. Multivariable analysis confirmed PNH positivity as an independent prognostic factor, in this subset of MDS and AA.

Finally, 190 patients were diagnosed with hemolytic PNH (cPNH) with a median clone size of 50% (38–99.9%) and some aplastic/dysplastic features (not diagnostic for AA or MDS) in 42 and 7% of cases. More than a half of them received eculizumab and ten cases were transplanted. As regards disease outcome, 24% of cases experienced at least one thrombotic event and 7% of them died, whilst death rate was lower at 2.8% in PNH cases without thrombosis (Supplementary Table 5).

## Discussion

In this large single-centre analysis over nearly 20 years, we confirm high prevalence of small and very small PNH clones in MDS and AA and provide evidence of their predictive and prognostic impact. In fact, we observed up to 1/5 PNH + MDS and 2/3 PNH + AA in our series, with 40% demonstrating clone size of <10%, and 30% showing <1% PNH clones. PNH positivity, at any clone size, was a good predictor of response to IST and of good outcome after HSCT in both diseases, and had a favorable impact on OS, with a dramatic reduction of the cumulative incidence of mortality even for clone size of 0.01%. Presence of PNH clones was correlated with thrombotic events, with a clear relationship with clone size, although other conventional risk factors for thrombosis have not been systematically evaluated. The latter, together with the presence of patients with thrombosis of unusual site in the cohort, were likely to account for the high prevalence of thrombosis in the PNH negative population.

The presence of PNH + MDS and AA is already known, although the rate of positivity varies among studies, mostly related to the different methods employed, the underlying diseases, and the threshold for positivity [1–7]. In fact, in a recent report evaluating 1004 individuals, the rate of PNH positivity increased from 4.5% in the pre-FLAER era to 9.2% in the FLAER one [4]. In BMFs, a prevalence of 4% was reported in adults with BMFs using a cutoff of 1% [1], and a positivity up to 40% was observed in children with a cutoff of 0.01% [2]. Finally, AA patients displayed larger clones than MDS, further confounding the prevalence data. In this study, we classified small clones as those <10%, the recognized threshold for subclinical PNH according to the International PNH Interest Group classification, and very small clones as those <1%, that are not even reported as positive by most laboratories. Our data (20% PNH+ in MDS, and 60% in AA) are in line with recent studies [1–3] and add the value of a population homogenously tested with FLAER at a single centre. A novel finding is the presence of PNH clones (mainly small and very small) in a number of not-commonly PNH-associated conditions: MDS-EB1/2, MPN, and ALs. Considering the former, we found a prevalence of about 15% correlating with better survival, whilst previous studies failed to detect PNH clones in this group [1, 5]. This may due to different methods employed (i.e., high-resolution 2-color flow cytometry in the study by Sugimori et al.) [5], variable cutoffs used in multi-center studies (i.e., 1 and 0.01% only in a fraction of patients in the study by Reza et al.) [1], and the possible presence of small fluctuating clones of uncertain significance. Similar considerations may be drawn for MPN and ALs, where PNH positivity may be transient, but not necessarily falsely positive. Even if some of the patients displayed clone sizes on granulocytes as high as 5% for MPN and 12% for AL, most subjects had a subclinical PNH clone with median clone size of 0.03% and 0.1% in MPN and AML, respectively. Although none of them developed classic hemolytic PNH, we demonstrated the persistency of PNH clones in 88% of the 26 cases that had been tested at least twice. Some Authors described the occurrence of PNH clones in MPN [13–15], and Fraiman et al. reported one patient developing a PNH clone of 60% on granulocytes and hypothesized the cooperation with CALR driver mutation in PNH clone selection and expansion [14]. Similar data were recently confirmed by Richards et al. in five patients with MPN that mainly showed association of PIG-A mutation and JAK2 V617F or other MPN driver mutations [13]. On the whole, the significance of smaller PNH clones, and the cutoff used to define positive/negative cases are largely debated, and one should probably refer to clinical or, at least, laboratory evidence hemolysis. Despite the different testing techniques over the years, cellular population analyzed, and sensitivity cutoffs used across studies, in our retrospective analysis, the largest proportion of patients (particularly AA and MDS) have been tested by FLAER and had a granulocyte clone size evaluable. For more unusual associations like MPN and AL, our results are to be taken cautiously, since the rarity and the paucity of the PNH clone and the abovementioned caveats necessitate prospective studies to clarify this issue. Finally, for these settings, *PIG-A* mutation analysis might add sensitivity to PNH clone detection.

We showed that PNH positivity is a good predictor of response to IST in both MDS and AA. The favorable impact on IST in AA has been described in several papers [7, 8, 16, 17]. At variance, a large study by Scheinberg et al. did not confirm this finding although a PNH clone size threshold of 1% was used [18]. The predictive value of PNH clones is even more uncertain in MDS with a favorable effect in an historical series not confirmed in recent studies [8, 18, 19]. The heterogeneity of the study populations and methods to detect PNH clones, may account for these discordant results. A novel finding of our study is the positive effect of small PNH clones on outcome after HSCT in both AA and MDS. Our series encompassed a large number of low-risk and hypoplastic MDS, showing clinical and laboratory features overlapping with AA (Fig. 2A, B), and possibly driving the therapeutic choice towards immunosuppressive approach. However, PNH positivity maintained its predictive value even in “higher risk” MDS patients, suggesting a favorable predictive role beyond marrow failure. Beside risk scores, favorable outcome after HSCT in PNH positive cases was expectedly associated with younger age. Furthermore, we report for the first time the impact of PNH positivity on OS of a large unselected population. Although this is partly due to the higher prevalence of PNH clones in diseases with better prognosis (i.e., AA rather than MDS and AML), and to eculizumab treatment in hemolytic PNH, PNH positivity retained a significant impact on OS also in multivariable analysis, and upon independently evaluating MDS and AA. As this is a retrospective data analysis, the presence of small PNH clones did not clinically drive the choice of HSCT in the present study, and OS outcomes were censored for HSCT. An independent control group of age-related MDS and AA would provide a better handle on this clinically challenging population and may be addressed in future studies.

Finally, in our series, the presence of PNH clones correlated with a trend for greater thrombotic risk in MDS, even in cases without overt hemolytic features. The pathogenesis of PNH related thrombosis is not fully understood and the association with clone size and intravascular hemolysis seems to weaken as recent evidences show high thrombotic risk even in non-hemolytic PNH patients (i.e., those with low LDH levels and moderate anemia). Griffin et al. showed that patients at higher risk of thrombosis were those with non-hemolytic PNH (i.e., LDH < 2xULN), high white cell clones and low red cell clones. Moreover, arterial thrombosis in this patient cohort was disproportionately high, and the Authors concluded that white cell and platelet factors have a higher role than previously thought in the mechanisms of thrombosis in PNH [9].

Notwithstanding the negative impact of PNH clones on thrombosis, we clearly demonstrated their positive predictive and prognostic value. This finding is difficult to explain, and we may speculate that PNH clones in AA and MDS represent “survivor populations” after an immunologic attack against marrow precursors, as already hypothesized in classic PNH [20]. The autoimmune pathogenesis is well established in AA [21], and hypothesized as a factor in low-risk MDS [8, 22], and the two diseases show great overlap of clinical and morphologic features. At the same time, the higher selective pressure of the immune system might exert stronger immunosurveillance on MDS evolution and leukemic progression that were significantly lower in PNH+ cases in our study.

Regardless of the postulated pathogenic role, our data suggest that systematic PNH testing might be a further tool to allow better prediction/prognostication and consequent clinical/laboratory follow-up in AA and MDS.

## Supplementary information

Supplementaty figures 1 to 11

Supplementary materials

Supplementary material for CD157 validation

Supplementary material for flow cytometric testing
