# Supplementary material for: Clinical and prognostic significance of small paroxysmal nocturnal hemoglobinuria clones in myelodysplastic syndrome and aplastic anemia
Source: Leukemia. 2021 Mar 4;35(11):3223–31. doi: 10.1038/s41375-021-01190-9 (PMC8550969; doi:10.1038/s41375-021-01190-9)
Supplement: Supplementary file 1 — Supplementaty figures 1 to 11 [file 41375_2021_1190_MOESM1_ESM.pdf]

Figure 1S

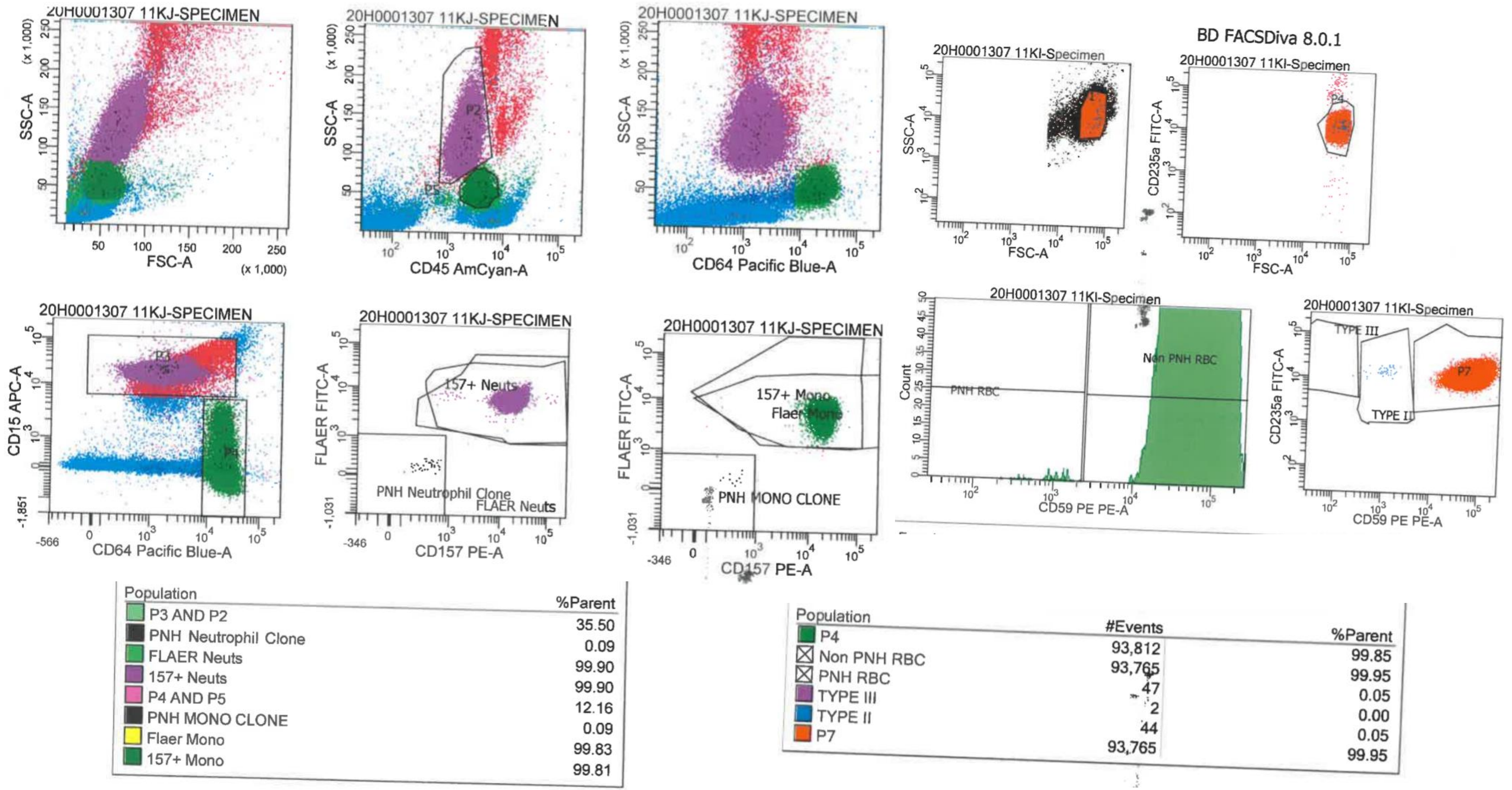

Population	%Parent
P3 AND P2	35.50
PNH Neutrophil Clone	0.09
FLAER Neuts	99.90
157+ Neuts	99.90
P4 AND P5	12.16
PNH MONO CLONE	0.09
Flaer Mono	99.83
157+ Mono	99.81

Population	#Events	%Parent
P4	93,812	99.85
Non PNH RBC	93,765	99.95
PNH RBC	47	0.05
TYPE III	2	0.00
TYPE II	44	0.05
P7	93,765	99.95

Figure 2S

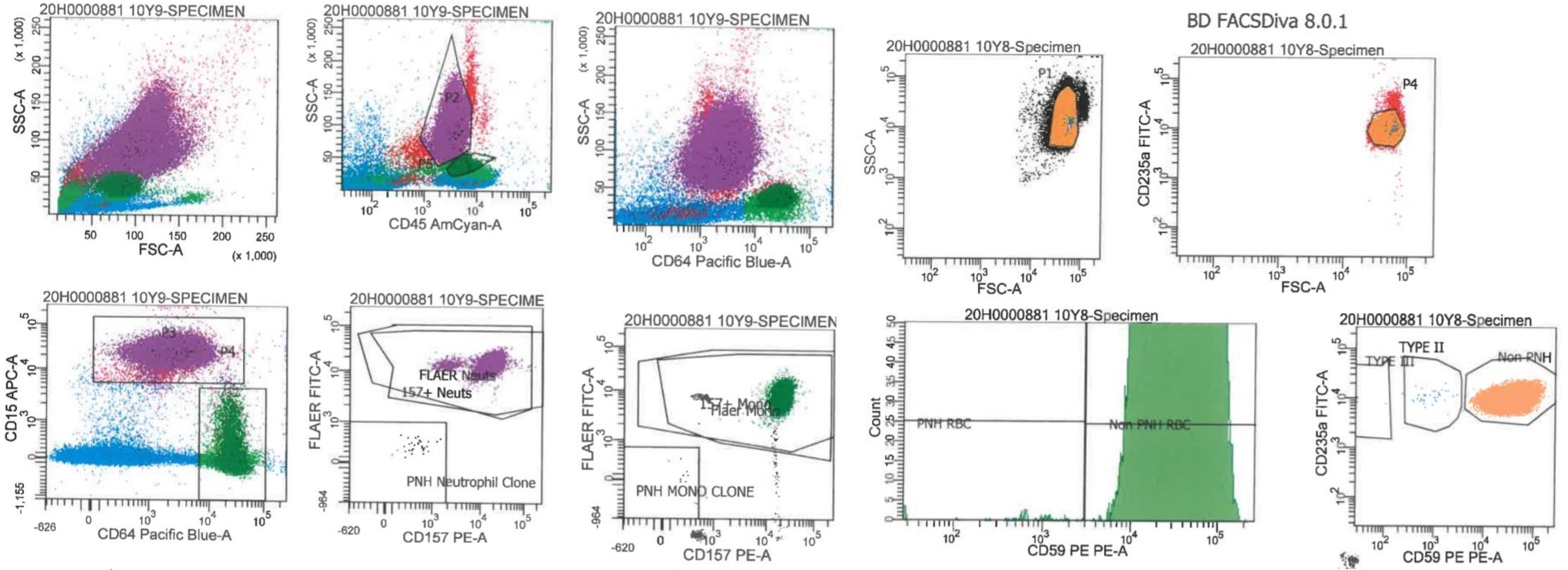

Population	%Parent
P3 AND P2	41.02
PNH Neutrophil Clone	0.06
FLAER Neuts	99.94
157+ Neuts	99.94
P4 AND P5	5.22
PNH MONO CLONE	0.11
Flaer Mono	99.88
157+ Mono	99.89

Population	#Events	%Parent
P4	111,002	98.96
Non PNH RBC	110,956	99.96
PNH RBC	46	0.04
TYPE III	5	0.00
TYPE II	39	0.04
Non PNH	110,954	99.96

Figure 3S

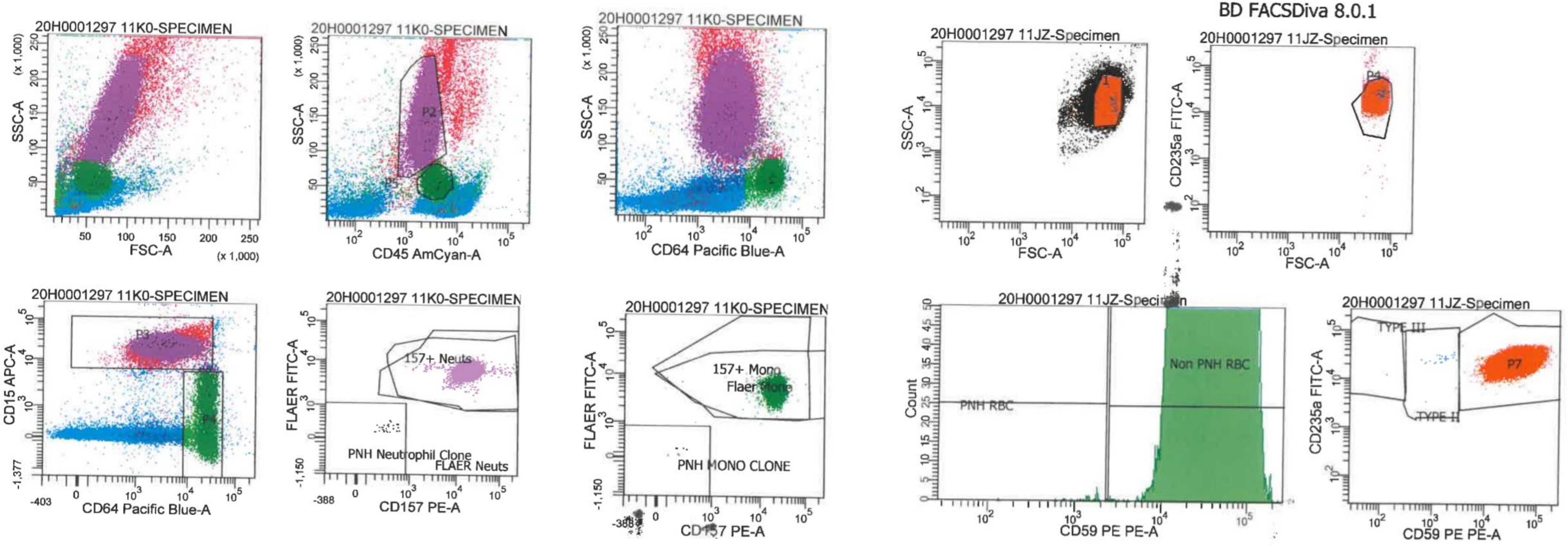

Population	%Parent
P3 AND P2	43.43
PNH Neutrophil Clone	0.06
FLAER Neuts	99.92
157+ Neuts	99.94
P4 AND P5	4.60
PNH MONO CLONE	0.13
Flaer Mono	99.79
157+ Mono	99.77

Population	#Events	%Parent
P4	93,970	99.60
Non PNH RBC	93,944	99.97
PNH RBC	24	0.03
TYPE III	1	0.00
TYPE II	29	0.03
P7	93,940	99.97

Figure 4S

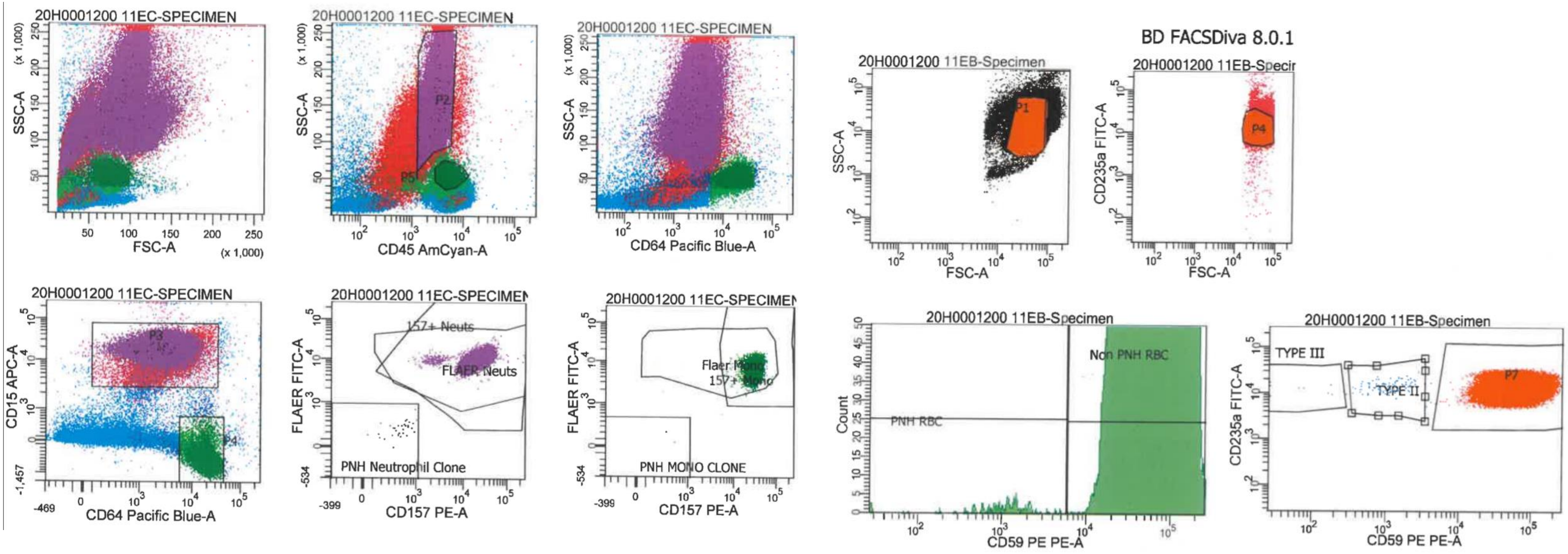

Population	%Parent
P3 AND P2	35.96
PNH Neutrophil Clone	0.04
FLAER Neuts	99.96
157+ Neuts	99.96
P4 AND P5	4.18
PNH MONO CLONE	0.02
Flaer Mono	99.89
157+ Mono	99.82

Population	#Events	%Parent
P4	462,159	95.30
Non PNH RBC	462,039	99.97
PNH RBC	120	0.03
TYPE III	10	0.00
TYPE II	103	0.02
P7	462,041	99.97

Figure 5S

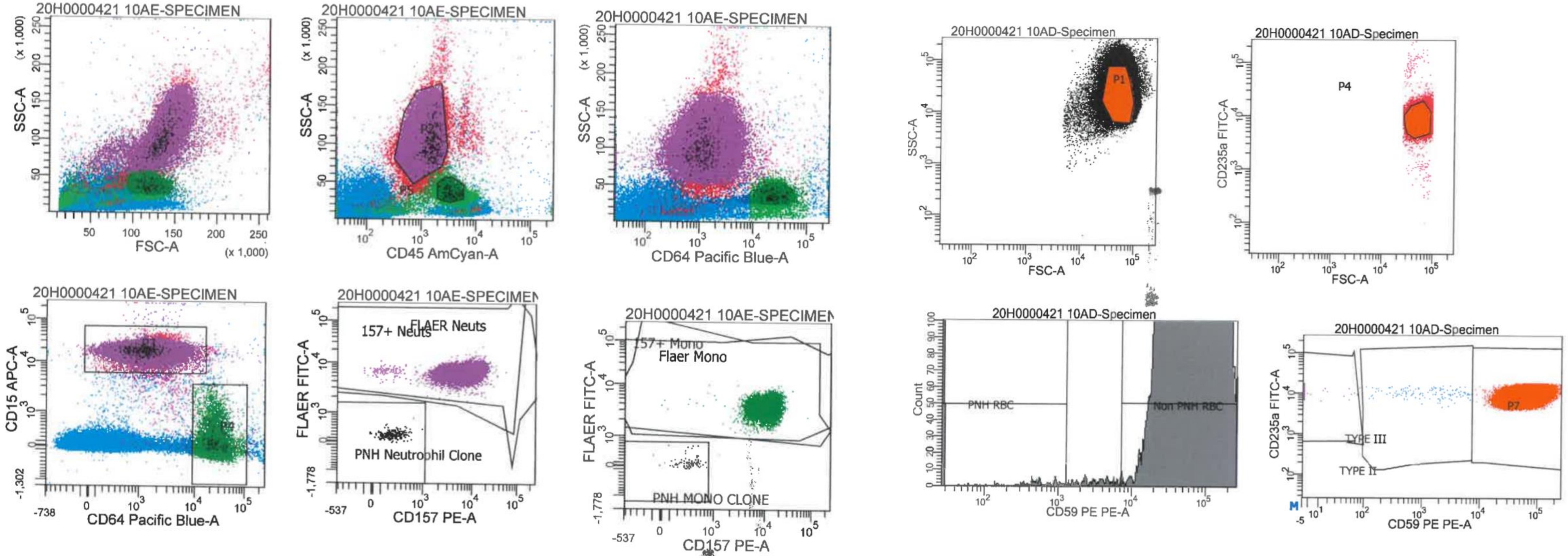

Population	%Parent
P3 AND P2	51.17
PNH Neutrophil Clone	0.21
FLAER Neuts	99.79
157+ Neuts	99.78
P4 AND P5	3.91
PNH MONO CLONE	0.90
Flaer Mono	99.08
157+ Mono	99.07

Population	#Events	%Parent
P4	89,637	86.80
Non PNH RBC	89,412	99.75
PNH RBC	159	0.18
TYPE III	98	0.11
TYPE II	128	0.14
P7	89,411	99.75

Figure 6S

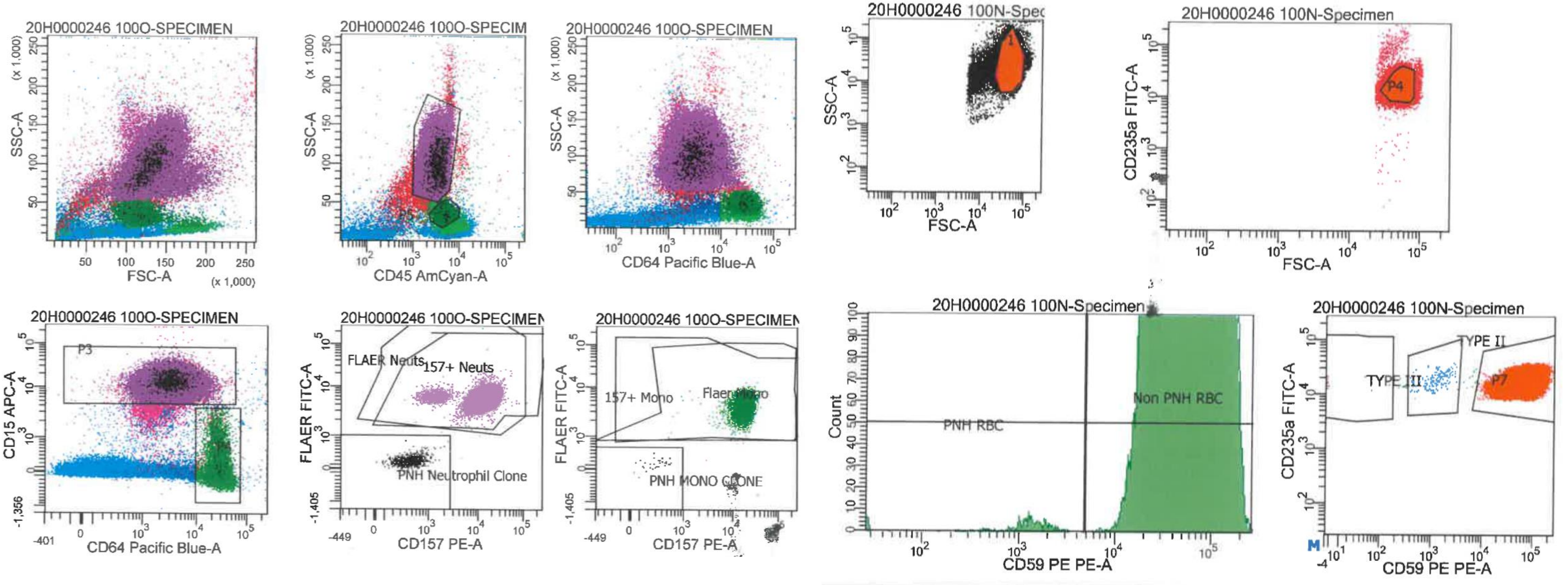

Population	%Parent
P3 AND P2	63.52
PNH Neutrophil Clone	0.48
FLAER Neuts	99.51
157+ Neuts	99.51
P4 AND P5	4.42
PNH MONO CLONE	0.35
Flaer Mono	99.56
157+ Mono	99.60

Population	#Events	%Parent
P4	129,128	93.15
Non PNH RBC	128,938	99.85
PNH RBC	190	0.15
TYPE III	10	0.01
TYPE II	170	0.13
P7	128,928	99.85

Figure 7S

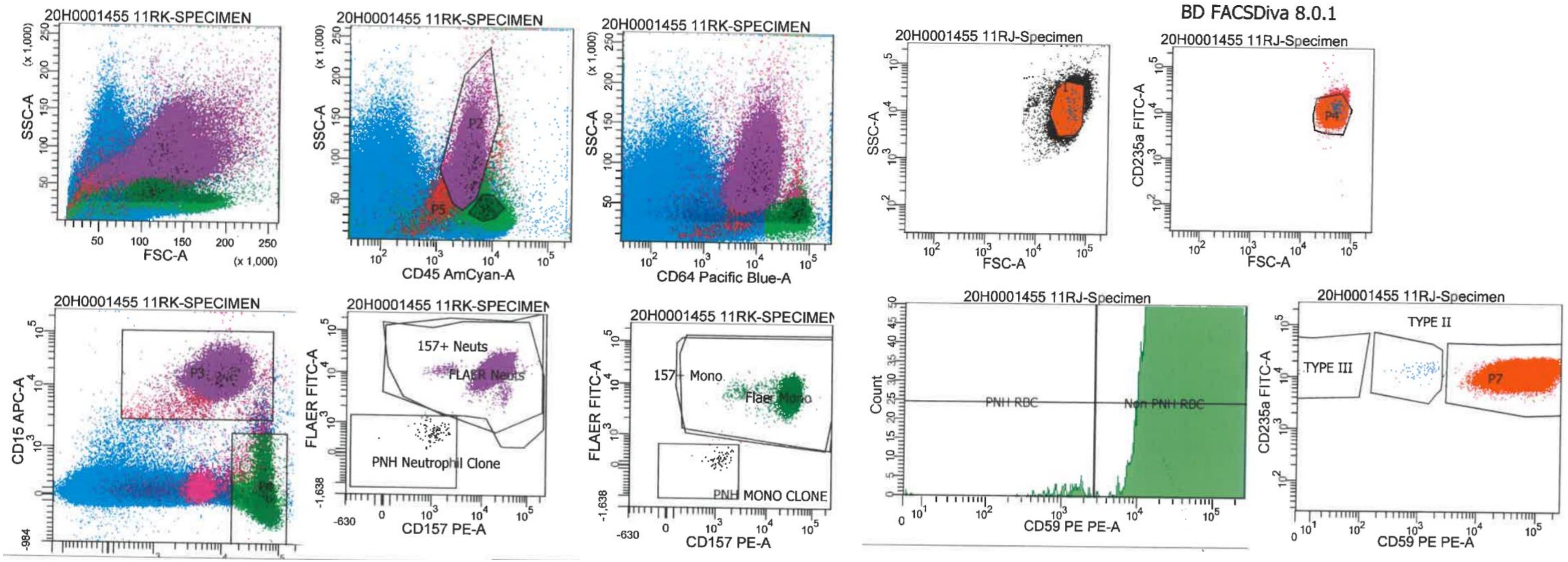

Population	%Parent
P3 AND P2	5.36
PNH Neutrophil Clone	0.13
FLAER Neuts	99.86
157+ Neuts	99.86
P4 AND P5	0.72
PNH MONO CLONE	0.63
Flaer Mono	99.31
157+ Mono	99.31

Population		%Parent
P4		99.20
Non PNH RBC	114,404	99.93
PNH RBC	77	0.07
TYPE III	3	0.00
TYPE II	74	0.06
P7	114,402	99.93

Figure 8S

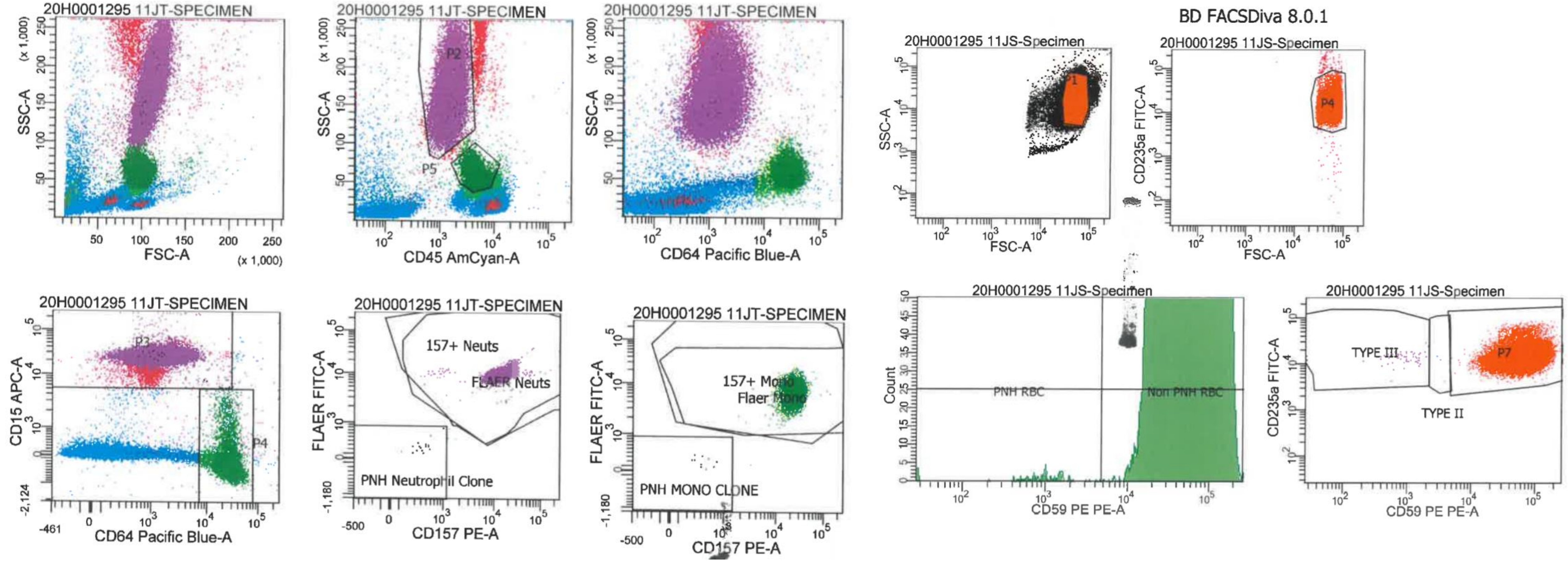

Population	%Parent
P3 AND P2	33.86
PNH Neutrophil Clone	0.06
FLAER Neuts	99.93
157+ Neuts	99.93
P4 AND P5	8.09
PNH MONO CLONE	0.15
Flaer Mono	99.84
157+ Mono	99.82

Population	#Events	%Parent
P4	131,095	99.86
Non PNH RBC	131,024	99.95
PNH RBC	71	0.05
TYPE III	66	0.05
TYPE II	4	0.00
P7	131,024	99.95

Figure 9S

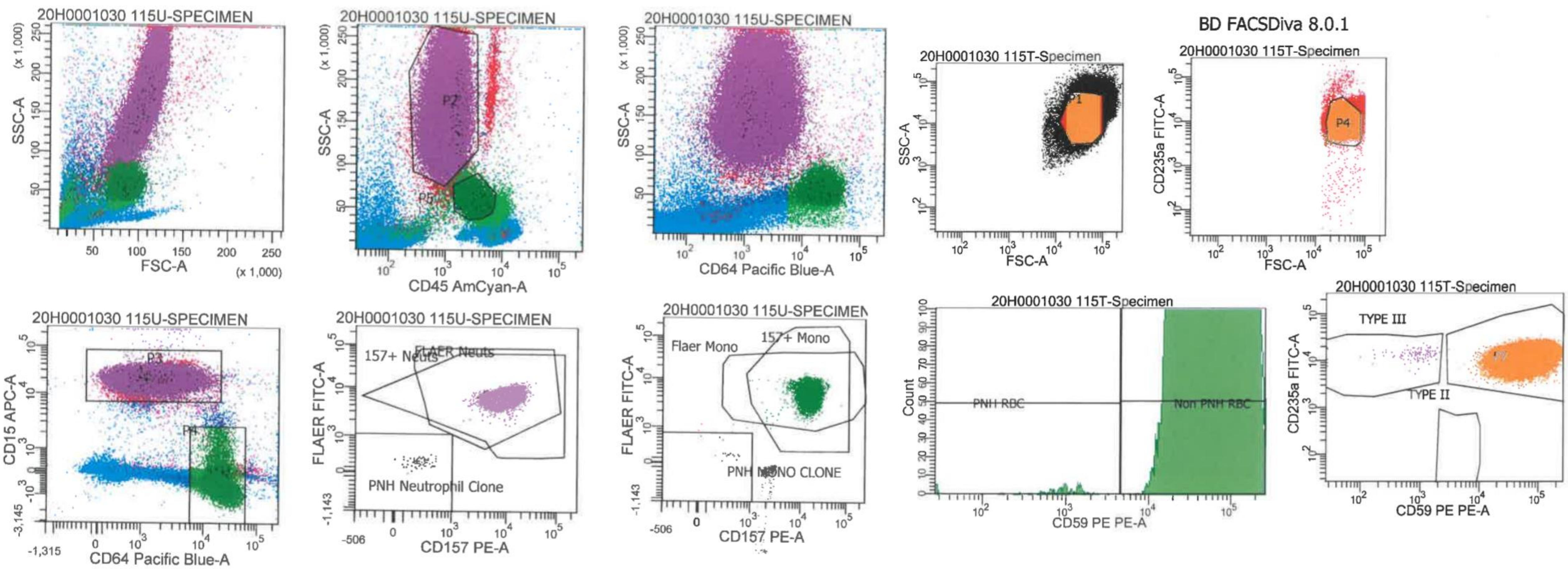

Population	%Parent
P3 AND P2	47.04
PNH Neutrophil Clone	0.08
FLAER Neuts	99.92
157+ Neuts	99.92
P4 AND P5	5.41
PNH MONO CLONE	0.15
Flaer Mono	99.76
157+ Mono	99.78

Population	#Events	%Parent
P4	442,048	76.56
Non PNH RBC	441,915	99.97
PNH RBC	132	0.03
TYPE III	130	0.03
TYPE II	0	0.00
P7	441,917	99.97

Figure 10S

Monocytes

Neutrophils

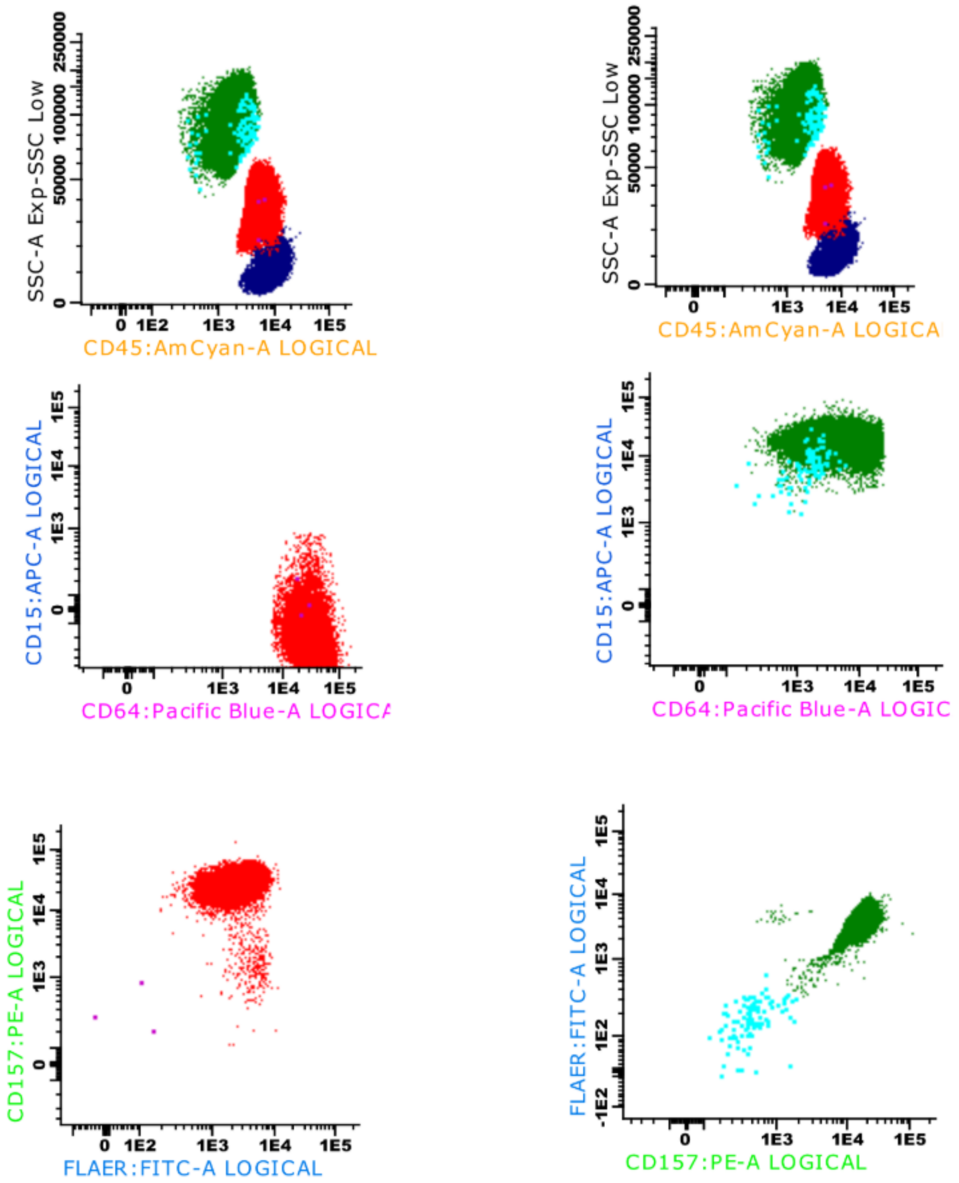

Common markers

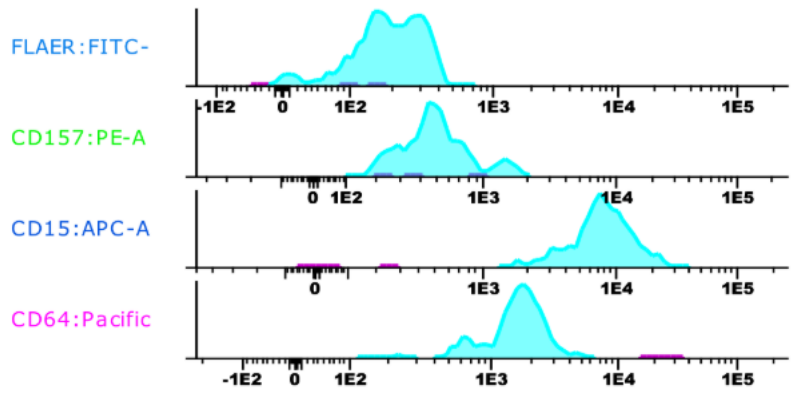

Sample Stability data

	All	All	All	Day 1	Day 1	Day 1	Day 3	Day 3	Day 3	Day 4	Day 4	Day 4	Day 5	Day 5	Day 5
	Total gated events	% of TNCs	% of pop	Total gated events	% of TNCs	% of pop	Total gated events	% of TNCs	% of pop	Total gated events	% of TNCs	% of pop	Total gated events	% of TNCs	% of p...
EVENTS	257582.00			257582.00											
NUCLEATED CELLS	202278.00	78.53 %		202278.00	78.53 %										
LYMPHOCYTES	87457.00	43.24 %		87457.00	43.24 %										
Monocytes	47817.00	23.64 %		47817.00	23.64 %										
FLAER+/CD157+ monos	47814.00	23.64 %		47814.00	23.64 %										
FLAER neg/PNH clone monos	3.00	0.00 %	0.01	3.00	0.00 %										
Granulocytes	46857.00	23.16 %		46857.00	23.16 %										
FLAER+/CD157+ grans	46753.00	23.11 %		46753.00	23.11 %										
FLAER neg/PNH clone grans	104.00	0.05 %	0.22	104.00	0.05 %	0.15									

Figure 11S

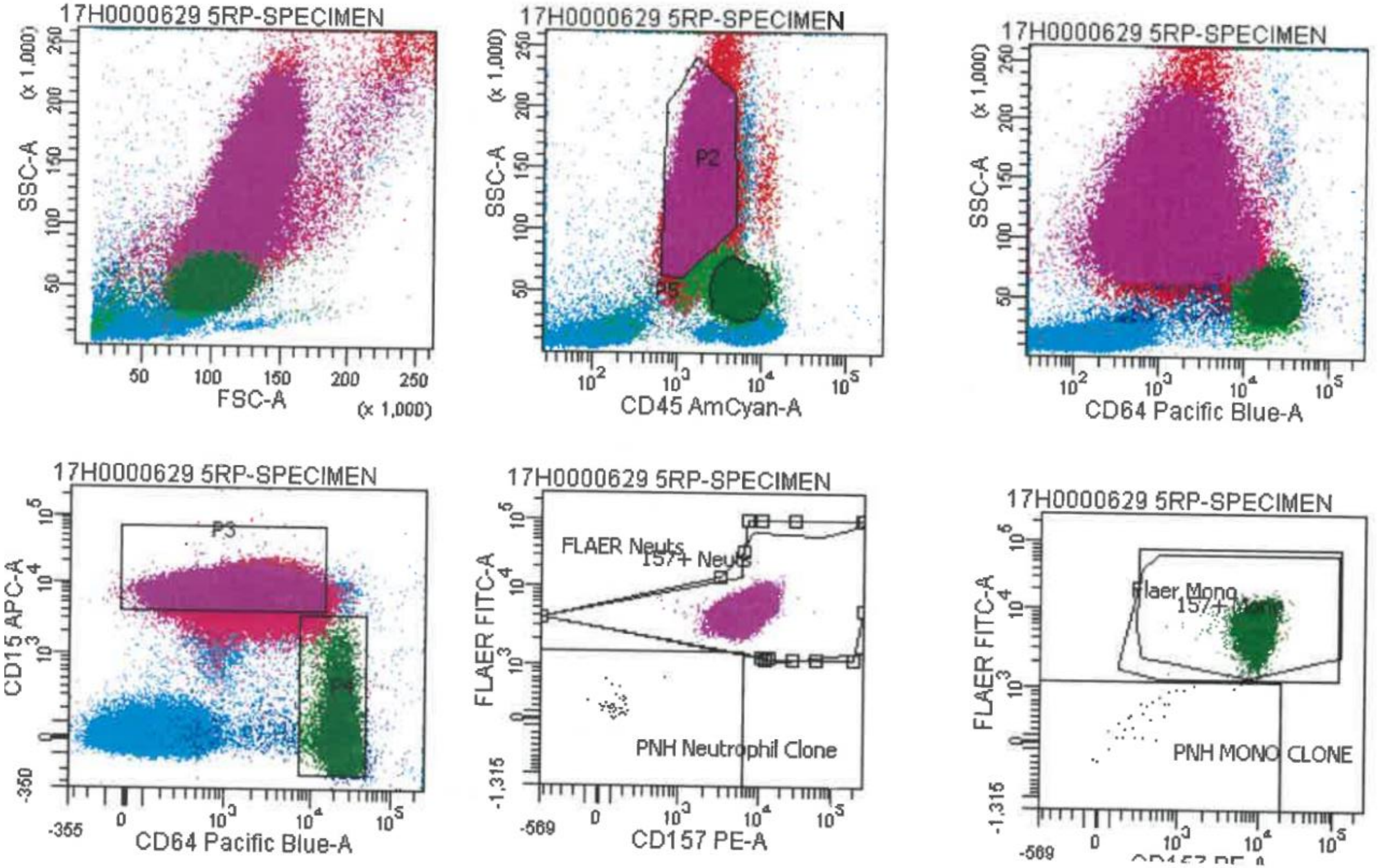

Population	#Events	%Parent	%Total
All Events	266,765	####	100.0
P3	191,053	71.6	71.6
P4	16,554	6.2	6.2
P5	14,679	5.5	5.5
P2	193,633	72.6	72.6
P3 AND P2	182,445	68.4	68.4
PNH Neutrophil Clone	40	0.0	0.0
FLAER Neuts	182,399	100.0	68.4
157+ Neuts	182,400	100.0	68.4
P4 AND P5	13,792	5.2	5.2
PNH MONO CLONE	29	0.2	0.0
Flaer Mono	13,752	99.7	5.2
157+ Mono	13,759	99.8	5.2

Population	%Parent
P3 AND P2	68.39
PNH Neutrophil Clone	0.02
FLAER Neuts	99.97
157+ Neuts	99.98
P4 AND P5	5.17
PNH MONO CLONE	0.21
Flaer Mono	99.71
157+ Mono	99.76
