# Supplementary material for: Clinical and prognostic significance of small paroxysmal nocturnal hemoglobinuria clones in myelodysplastic syndrome and aplastic anemia
Source: Leukemia. 2021 Mar 4;35(11):3223–31. doi: 10.1038/s41375-021-01190-9 (PMC8550969; doi:10.1038/s41375-021-01190-9)
Supplement: Supplementary file 2 — Supplementary materials [file 41375_2021_1190_MOESM2_ESM.doc]

**Table 1S**

**Patients evaluated at least twice divided according to clone size dynamics**

|  | **Declining N=62** | **Stable N=94** | **Increasing N=74** |
| --- | --- | --- | --- |
| **Males, N(%)** | 29 (47) | 46 (49) | 36 (49) |
| **Females, N(%)** | 33 (53) | 48 (51) | 38 (51) |
| **Median age, years (IQR)** | 43 (30-68)** | 53 (28-70) | 44 (29-81) |
| **MDS, N(%)** | 15 (23) | 24 (26) | 12 (16) |
| **AA, N(%)** | 29 (47) | 40 (43) | 38 (51) |
| **MDS/AA, N(%)** | 2 (3) | 0 (0) | 3 (4) |
| **Acute leukemia, N(%)** | 1 (2) | 4 (4) | 0 (0) |
| **Hemolytic PNH, N(%)** | 13 (21) | 3 (3)* | 20 (27) |
| **MPN, N(%)** | 0 (0) | 3 (3) | 0 (0) |
| **MDS/MPN, N(%)** | 0 (0) | 1 (1) | 0 (0) |
| **Isolated cytopenia, N(%)** | 0 (0) | 10 (11) | 1 (1) |
| **Isolated thrombosis, N(%)** | 1 (2) | 4 (4) | 0 (0) |
| **Others, N(%)** | 1 (2) | 5 (5) | 0 (0) |
| **Hb<100 g/L, N(%)** | 30 (48) | 36 (38) | 29 (39) |
| **PLT<100x10^9^/L, N(%)** | 38 (61) | 40 (43) | 40 (54) |
| **ANC<1.5x10^9^/L, N%** | 31 (50) | 40 (43) | 32 (43) |
| **Median LDH, U/L (range)** | 257 (97-3413) | 227 (83-1851) | 288 (83-443) |
| **IST, N(%)** | 28 (45) | 39 (42) | 33 (45) |
| **Chemotherapy, N(%)** | 2 (3) | 9 (10) | 0 (0) |
| **HSCT, N(%)** | 12 (19) | 18 (19) | 15 (20) |
| **Eculizumab, N(%)** | 15 (24) | 6 (6) | 27 (37)* |
| **MDS Progression, N(%)** | 0 (0) | 4 (4) | 1 (1) |
| **AML evolution, N(%)** | 1 (2) | 0 (0) | 1 (1) |
| **Thrombosis, N(%)** | 11 (18) | 13 (14) | 8 (11) |
| **Death, N(%)** | 10 (16) | 14 (15) | 8 (11) |

*p<0.0001 versus both; **p=0.02 vs stable

Fluctuations were defined as increase/declining of granulocyte PNH clone size >5%. MDS myelodysplastic syndromes, AA aplastic anemia, MDS/AA hypoplastic MDS, MPN myeloproliferative neoplasms. IST immunosuppressive therapy, HSCT hematopoietic stem cell transplant. Hb hemoglobin; PLT platelets; ANC absolute neutrophil counts.

| **Table 2S**  **Clinical and laboratory baseline characteristics, treatment and outcome of MDS Patients screened for PNH clones.** | | |
| --- | --- | --- |
|  | **PNH neg N=693 (79.7%)** | **PNH pos N=176**  **(20.3%)** |
| **Males, N(%)** | 424 (61) | 91 (52)* |
| **Females, N(%)** | 269 (39) | 85 (48)* |
| **Median age, years (IQR)** | 63 (53-70) | 59 (48-67)* |
| **Constitutional MDS, N(%)** | 7 (1) | 0 (0) |
| **RCMD, N(%)** | 268 (39) | 55 (31) |
| **RCUD, N(%)** | 3 (0.4) | 0 (0) |
| **RA, N(%)** | 28 (4) | 4 (2) |
| **RA del5q, N(%)** | 23 (3) | 3 (2) |
| **HypoMDS, N(%)** | 90 (13) | 67 (38)** |
| **EB1/2, N(%)** | 199 (29) | 36 (21)* |
| **RARS/RARS-T, N(%)** | 34 (5) | 3 (2) |
| **Fibrotic MDS, N(%)** | 3 (0.4) | 0 (0) |
| **t-MDS, N(%)** | 11 (2) | 1 (0.6) |
| **ICUS, N%** | 27 (4) | 7 (4) |
| **Hematologic values and disease specific score** | **N=693** | **N=176** |
| **Hb<100 g/L, N(%)** | 258 (37) | 81 (46)* |
| **PLT<100x109/L, N(%)** | 272 (39) | 106 (60)** |
| **ANC<1.5, N(%)** | 320 (46) | 94 (53) |
| **Median LDH, U/L (range)** | 193 (73-1864) | 233 (133-1520)** |
| **Median Blasts, %(range)** | 3 (0-19) | 3 (0-19) |
| **monosomy 7, N(%)** | 28 (4) | 14 (8)* |
| **complex karyotype, N(%)** | 44 (6) | 6 (3) |
| **IPSS low, N(%)** | 281 (41) | 59 (33) |
| **IPSS int1, N(%)** | 237 (34) | 85 (48)** |
| **IPSS int2, N(%)** | 147 (21) | 22 (12)* |
| **IPSS high, N(%)** | 28 (4) | 10 (6) |
| **IPSS-R very low, N(%)** | 28 (4) | 9 (5) |
| **IPSS-R low, N(%)** | 201 (29) | 72 (41) |
| **IPSS-R int, N(%)** | 229 (33) | 47 (27) |
| **IPSS-R high, N(%)** | 166 (24) | 30 (17) |
| **IPSS-R very high, N(%)** | 69 (10) | 18 (10) |
| **Outcome** | **N=693** | **N=176** |
| **MDS Progression, N(%)** | 34 (5) | 1 (0.6)* |
| **AML evolution, N(%)** | 88 (13) | 12 (7)* |
| **Thrombosis, N(%)** | 37 (5) | 16 (9) |
| **Death, N(%)** | 308 (44) | 48 (27)** |
| **Treatments** | **N=365 (52.6%)** | **N= 108 (61.3%)** |
| **Chemotherapy, N(%)** | 191 (52) | 29 (27)** |
| **Cyclosporine, N(%)** | 40 (11) | 37 (34)** |
| **ATG, N(%)** | 10 (3) | 13 (12)** |
| **Azacytidine, N(%)** | 123 (34) | 19 (18)* |
| **Eltrombopag, N(%)** | 8 (2) | 5 (5) |
| **HSCT, N(%)** | 141 (39) | 40 (37) |
| **Eculizumab, N(%)** | 0 (0) | 7 (6)** |

*p<0.01; **p<0.0001. RCMD refractory cytopenia with multilineage dysplasia, RCUD refractory cytopenia with unilineage dysplasia, RA refractory anemia, hypoMDS hypoplastic myelodysplastic syndrome, EB MDS with excess of blasts, RARS/RARS-T refractory anemia with ring sideroblasts with or without thrombocytosis, fibrotic MDS myelodysplasia with marrow fibrosis, t-MDS therapy related, ICUS idiopathic cytopenia with of uncertain significance, ATG anti-thymocyte globulin, HSCT hematopoietic stem cell transplant Hb hemoglobin; PLT platelets; ANC absolute neutrophil counts.

**Table 2.1S: PNH prevalence and clone size in patients with MDS with excess of blasts 1 or 2 (MDS-EB1/2), myeloproliferative neoplasms (MPN), MPN/MDS and with acute leukemia (AL).** *p=0.03

**PNH+ patients with MPN (N=16, 17%) belonged to the following categories: myelofibrosis N=8, chronic myeloid leukemia N=2, polycythemia rubra vera N=1, MPN unclassified N=5. PNH+ patients with AL (N=29, 12%) were acute lymphoblastic leukemia N=2, and acute myeloid leukemia N=27. All patients had been tested at diagnosis as part of the initial workup of anemia with LDH elevation (8 MPN, 5 MDS/MPN, and 17 AL with standard technique with sensitivity >0.05% and 8 MPN, 4 MDS/MPN, and 12 AL by FLAER technique). Seventeen patients with MPN or MPN/MDS, 9 with AL, and 6 with MDS-EB1/2 had been tested twice and PNH positivity was confirmed in all but 3 MPN cases.**

| **MPN and AL** | **PNH+ MPN, N=16 (17%) and PNH+ MDS/MPN, N=9 (9%)** | **PNH+ AL**  **N=29 (12%)** |
| --- | --- | --- |
| **Median clone size %(range)**  **Granulocytes**  **Monocytes**  **Erythrocytes** | **0.03(0.01-3)**  **0.1 (0.02-5)**  **0.1(0.03-0.6)** | **0.1(0.01-12.4)**  **0.2 (0.02-8.4)**  **0.1(0.05-3.7)** |
| **MDS-EB1/2** | **PNH positive N=36 (15%)** | **PNH negative N=199 (85%)** |
| **Median clone size %(range)**  **Granulocytes**  **Monocytes**  **Erythrocytes** | **0.3(0.01-20.7)**  **0.1(0.02-17.7)**  **0.1(0.03-2.44)** | **-**  **-**  **-** |
| **Therapies performed** | | |
| **Chemotherapy, N(%resp)**  **IST, N(%resp)**  **Azacytidine, N(%resp)**  **HSCT, N(%resp)** | **14 (43)**  **1 (0)**  **18 (33)**  **10 (60)** | **104 (50)**  **3 (33)**  **80 (41)**  **64 (78)** |
| **Complications and outcome** | | |
| **Thrombosis, N(%)**  **AML evolution, N(%)**  **Dead, N(%)**  **Median OS, months (range)** | **2 (6)**  **9 (25)**  **19 (54)**  **16 (6-134)** | **12 (6)**  **66 (33)**  **153 (77)***  **17 (2-121)** |

**IST immunosuppressive therapy, HSCT hematopoietic stem cell transplant.**

| **Table 2.2S: PNH prevalence and clone size in patients with hypoplastic myelodysplastic syndromes (MDS).** This category included those with bone marrow cellularity <25% and/or considered low after adjustment for age. *p<0.01   | **Hypoplastic MDS** | **PNH positive N=67 (43%)** | **PNH negative N= 90 (57%)** | | --- | --- | --- | | **Median clone size %(range)**  **Granulocytes**  **Monocytes**  **Erythrocytes** | **0.39 (0.01-86)**  **0.3(0.1-99)**  **0.1(0.2-37)** | **-**  **-**  **-** | | **Hb g/L, median (range)**  **Plt x10^9/L, median(range)**  **Neutrophils x10^9/L, median (range)**  **LDH UI/L, median (range)** | **102 (68-153)***  **40 (5-576)***  **1,36(0,04-5,8)**  **219 (133-1553)*** | **108 (65-165)**  **111 (8-727)**  **1,59 (0,02-8,4)**  **186 (92-814)** | | **Therapies performed** | | | | **Chemotherapy, N(%resp)**  **IST, N(%resp)**  **Azacytidine, N(%resp)**  **HSCT, N(%resp)** | **3 (4)**  **28 (42)***  **0 (0)**  **13 (19)** | **12 (13)**  **18 (20)**  **7 (8)***  **13 (14)** | | **Complications and outcome** | | | | **Thrombosis, N(%)**  **AML evolution, N(%)**  **Dead, N(%)**  **Median OS, years (range)** | **5 (7.5)**  **1 (1)**  **19 (28)**  **2.8 (1-10.3)*** | **4 (4)**  **3 (3)**  **35 (39)**  **1.6 (0.9-9.7)** |   IST immunosuppressive therapy, HSCT hematopoietic stem cell transplant. Hb hemoglobin; PLT platelets;  **Table3S Clinical and laboratory baseline characteristics, treatment and outcome of aplastic anaemia (AA) patients screened for PNH clones.** | | | |
| --- | --- | --- | --- | --- | --- | --- | --- | --- | --- | --- | --- | --- | --- | --- | --- | --- | --- | --- | --- | --- | --- | --- | --- | --- |
|  | **PNH neg=204**  **(38.4%)** | **PNH pos N=327**  **(61.6%)** |  |
| **Males, N(%)** | 107 (53) | 167 (51) |  |
| **Females, N(%)** | 97 (48) | 160 (49) |  |
| **Median Age, years (IQR)** | 46 (33-61) | 43 (29-59) |  |
| **Overlapping dysplasia, N(%)** | 10 (5) | 3 (1)* |  |
| **AA type**  **Moderate, N(%)**  **Severe, N(%)**  **Very severe, N(%)** | 104 (51)  47 (23)  53 (26) | 209 (64)  82 (25)  36 (11) |  |
| **Hematologic values** | **N=204** | **N=327** |  |
| **Hb<100 g/L, N(%)** | 107 (53) | 158 (48) |  |
| **PLT<100x10^9^/L, N(%)** | 6 (3) | 241 (74)** |  |
| **ANC<1.5x10^9^/L, N(%)** | 121 (59) | 198 (61) |  |
| **Median Ret, x10^9^/L, (range)** | 44 (3-422) | 54 (1-554)* |  |
| **Median LDH, U/L (range)** | 187 (78-748) | 223 (70-3102)** |  |
| **monosomy 7, N(%)** | 5 (3) | 8 (2) |  |
| **Other karyo alterations, N(%)** | 6 (5) | 20 (6) |  |
| **Treatments** | **N=161 (78.9)** | **N=323 (98.8)** |  |
| **ATG, N(%)** | 72 (45) | 245 (76)** |  |
| **Cyclosporine, N(%)** | 97 (60) | 273 (84)** |  |
| **Androgen, N(%)** | 10 (6) | 3 (1)* |  |
| **Eltrombopag, N(%)** | 11 (7) | 23 (7) |  |
| **HSCT, N(%)** | 55 (34) | 82 (25)* |  |
| **Eculizumab, N(%)** | 0 (0) | 48 (15)** |  |
| **Outcome** | **N=204** | **N=327** |  |
| **MDS Progression, N(%)** | 14 (7) | 7 (2)* |  |
| **AML evolution, N(%)** | 6 (3) | 6 (2) |  |
| **Thrombosis, N(%)** | 11 (5) | 21 (6) |  |
| **Death, N(%)** | 70 (34) | 38 (12)** |  |

*p<0.01; **p<0.0001. Ret reticulocytes, ATG anti-thymocyte globulin, HSCT hematopoietic stem cell transplant. Hb hemoglobin; PLT platelets; ANC absolute neutrophil counts.

**Table 4S**

**Predi**ctors of OS by multivariable analysis

| **variable** | **Hazard Ratio** | **95% Conf. Interval** |
| --- | --- | --- |
| **Male gender** | 1.36 | 1.14 – 1.63 |
| **Age >40 years** | 3.14 | 2.34 – 4.21 |
| **Treated cases** | 1.45 | 1.18 – 1.78 |
| **PNH negativity** | 2.28 | 1.18-4.4 |
| **Diagnosis of AA versus MDS** | 0.61 | 0.48 – 0.77 |
| **Diagnosis of classic PNH versus MDS** | 0.11 | 0.04 – 0.3 |
| **Diagnosis of acute leukemia versus MDS** | 1.35 | 1 – 1.84 |
| **Other diagnosis versus MDS** | 0.66 | 0.13 – 1.13 |

**Table 5 S**

**Clinical and hematologic characteristics of patients with hemolytic PNH**

| **Number of pts tested** | 190 |
| --- | --- |
| **Males, N(%)** | 87 (46) |
| **Females, N(%)** | 103 (54) |
| **Median age, years (IQR)** | 43 (22-67) |
| **dysplastic features, N(%)** | 14 (7) |
| **hypoplastic features, N(%)** | 79 (42) |
| **Hb<100 g/L, N(%)** | 100 (53) |
| **PLT<100x10^9^/L, N(%)** | 70 (37) |
| **ANC<1.5x10^9^/L, N(%)** | 54 (28) |
| **Median LDH U/L, (range)** | 577 (118-4614) |
| **Thrombosis, N(%)** | 45 (24) |
| **Death, N(%)** | 14 (7) |
| **Eculizumab, N(%)** | 129 (68) |
| **HSCT, N(%)** | 10 (5) |

MDS myelodysplastic syndromes, AA aplastic anemia, HSCT hematopoietic stem cell transplant. Hb hemoglobin; PLT platelets; ANC absolute neutrophil counts.

**Supplementary Figure Legends**

**Supplementary figures: Flow cytometer plots of very small PNH clones in 11 representative patients** in different disease categories; myelodysplastic syndromes (MDS) with multilineage dysplasia (MDS-MLD Figure 1S), hypoplastic MDS (Figure 2S), MDS with single lineage dysplasia (MDS-SLD, Figure 3S), MDS with ring sideroblasts (MDS-SLD-RS, Figure 4S), MDS with excess of blasts type 1 (MDS-EB1, Figure 5S), aplastic anemia (AA, Figure 6S), myeloproliferative syndrome unclassifiable (MPN-U, Figure 7S), MDS/MPN overlap (Figure 8S), acute myeloid leukemia (AML de novo Figure 9S and AML evolved from MDS-EB2 Figure 10S), and myeloproliferative syndrome (primary myelofibrosis, Figure 11S).

**Supplementary methods**

Since 2015, we have used CD157 for PNH cells testing and validated it since 2017 (See also the Standard Operating Procedure document on PNH testing and the validation report on CD157). CD157 is another GPI linked structure expressed on both granulocytes and monocytes. ICCS have published data on CD157 and its ability to replace CD24 and CD14 in predicate 4 color granulocyte and monocyte assays respectively. ICCS 2013 recommends single tube assay to monitor both granulocytes and monocytes by using CD157. We have used the CD157 since 2015 and this has been validated since 2017 in our laboratory (see attached Standard Operating Procedure document on PNH testing and validation report on CD157). The 5-color reagent tube includes with the combination of FLAER, CD157, CD45, CD15 and CD64. While CD157 based 4 and 5 color assays generated closely similar results to the predicate assays on a range of PNH and normal samples, the 5-color assay has significant advantages. Only a single 5 color WBC reagent cocktail is required to detect both PNH granulocytes and monocytes. Using 5 color single tube has further additional advantage on sample preparation and analysis time is reduced yielding significant efficiencies in technical resources and reagent costs. These 5 color cocktail reagents with CD157 for both granulocytes and monocytes are monitored by UK NEQAS EQA scheme and the laboratory has excellent correlation on all reports in line with consensus reports.

As per the ICCS/ESCAA recommendations (Guidelines to detect GPI deficient cells in PNH Part2- reagent selection and Assay optimization for High sensitivity testing, Sunderland et al 2017), two GPI linked structures are analysed per WBC lineage assessed. Combinations of reagents CD157, CD15 and FLAER represent the most tested combination of reagents to detect GPI-deficient neutrophils, while FLAER in combination with CD157 and CD64 represent the most validated combination of reagents to detect GPI-deficient monocytes.

In the past CD33 was widely used to gate both neutrophils and monocytes. However, there are several drawbacks to this approach. On difficult sample types, for example MDS, CD33 is usually inferior to CD15 for gating neutrophils. Furthermore, CD33 fails to delineate platelet aggregates which present from mature neutrophils and such ‘contaminants’ can mimic PNH neutrophils on FLAER versus CD24 plots. Additionally, CD33 expression in some rare cases can be very low or sometimes it can be negative. CD33 is also not effective at delineating monocytes from basophils and it can be mimic as PNH monocytes due to their low binding of FLAER and lack of staining with CD14. In contrast, CD64 is brightly expressed on monocytes but is not expressed on basophils. If myeloid blasts are present, it can mimic monocytes in their expression of CD33 and such cells are also difficult to delineate from PNH monocytes.

CD157 is a GPI linked structure brightly expressed on both neutrophils and monocytes and cytometers in our lab is configured with >5 PMTs and therefore we use 5 color combination with FLAER, CD157, CD15, CD64 and CD45 instead of CD24 and CD14 for single tube high sensitivity assessment of GPI deficient neutrophils and monocytes.

The gating strategy used are as follows.


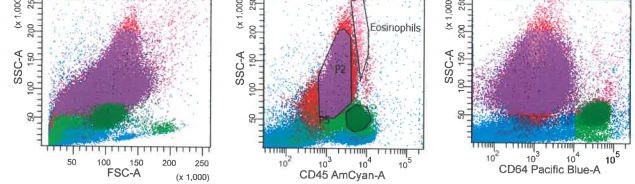


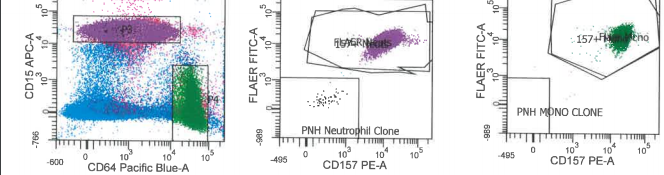


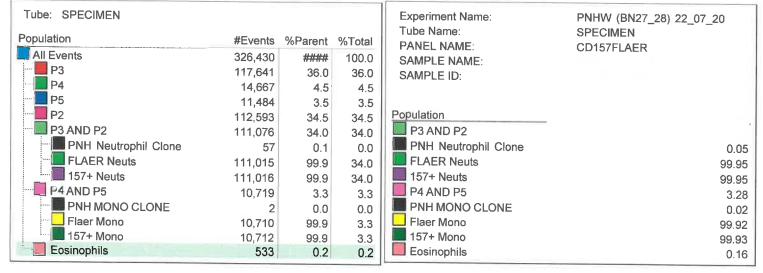


1. Plot 1 SSA-A/FSC-A
2. Plot 2 SSA-A/CD45 AmCyan-A. Gated neutrophils as P2 and Eosinophils excluded (by invert gate) CD45 bright, high SSC characteristics. Monocytes gated as P5.
3. Plot 4 (a) CD15 APC-A versus CD64 BV421-A. Gated high expression of CD15 in P3 gate from P2 gate (which already excluded Eosinophils by invert gate). By this way, we can completely eliminate eosinophil contamination in P3 gate

(b) Monocyte gate (P4) from plot 4 (1^st^ plot from second row) against bright CD64 expression which are negative for CD15(if normal monocytic cells).

1. Plot 5 (middle plot on 2^nd^ row) gated versus FLAER and CD157.
2. FLAER positive CD157+ neutrophils and PNH clones/GPI deficient cells (FLAER- CD157-) gated from P2 and P3 on one gate. This strategy allows to include only high CD15 neutrophils and exclude eosinophils.


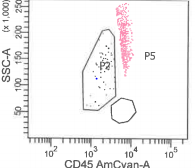

This plot back gated to show where GPI deficient cells falling on P2 gate and eosinophils (excluded from neutrophils) on CD45/SSA-A based on SSC expression and CD15 expression(Eosinophils are with low CD15 expression).

1. FLAER+ CD157+ monocytes and PNH clones/GPI deficient cells(FLAER- CD157-) gated from P4 and P5 (monocytic gates based on CD64, CD15 and CD45- 1^st^ plot from 2^nd^ row)
