# Supplementary material for: Clinical and prognostic significance of small paroxysmal nocturnal hemoglobinuria clones in myelodysplastic syndrome and aplastic anemia
Source: Leukemia. 2021 Mar 4;35(11):3223–31. doi: 10.1038/s41375-021-01190-9 (PMC8550969; doi:10.1038/s41375-021-01190-9)
Supplement: Supplementary file 3 — Supplementary material for CD157 validation [file 41375_2021_1190_MOESM3_ESM.pdf]

ASSAY REVALIDATION REPORT

Validation of Beckton Dickinson (BD) CD157 PE for immediate use in the PNH assay

QR-HAE-VR0000200

Report Prepared by: Katy Sanchez

Date: 05/09/2017

On completion this document will constitute the Revalidation Report, and should be read in conjunction with the corresponding Revalidation Plan.
For revalidations where particular sections do not apply, these should be completed with 'not applicable' as the entry

1. CONTENTS PAGE

Section

1 Contents

2 Reason for revalidation

3 Work risk assessment / COSHH changes

4 Assessment of EQA performance

5 Assay parameter review

6 Review of Results against Acceptance Criteria

7 Problem report

8 Documentation review

9 Report Summary and Recommendations

10 Revalidation Authorisation Associated Bibliography

11 Bibliography

2. REASON FOR REVALIDATION

Due to a supplier issue (see QE404), the regular order of CD157 PE (clone SY11B5) from 'The Binding Site' (manufactured by 'eBioscience') was not delivered leaving the laboratory very short of reagent. Correspondence with 'The Binding Site' revealed that they were no longer able to supply this reagent and we would need to find alternative distributer/manufacturer.

Contact was made with Beckton Dickinson (BD) who also manufactures a CD157 PE (clone SY11B5) and email correspondence was made with Robert Sutherland, field expert, who was lead author for the CD157/FLAER-based assay in use within the laboratory (Sutherland et al, 2014). Robert had also previously tested CD157 from BD and reported similar expression to the eBioscience reagent giving us confidence that a switch of manufacturer from eBioscience to BD would be acceptable following validation.

Due to the very short notice period and limited stock supply whilst maintaining a full patient PNH service, a formal validation plan was not achievable. All validation information is provided in this document (or attached to the Q-Pulse file). (Remaining stock only allowed for 14 samples to be tested in parallel. Some reagent also sent to the Royal London Hospital Immunophenotyping Laboratory for validation – to be attached separately if supplied).

3. WORK RISK ASSESSMENT / COSHH CHANGES

Not applicable, no change to work processes or COSHH. Reagent constituants remain the same, only a change in manufacturer.

4. ASSESSMENT OF EQA PERFORMANCE

EQA material received and tested as part of the validation.

5. ASSAY PARAMETER REVIEW

Antibody performance was assessed by running samples in parallel with the existing antibody by testing correlation and linearity.

	MONOS						NEUTS					
	% Mono clone		Median Fluorescence		SI		% Neut clone		Median Fluorescence		SI	
Lab no	eBio	BD	eBio	BD	eBio	BD	eBio	BD	eBio	BD	eBio	BD
17h3897	61.86	61.99	179.17	147.36	83.96	84.73	64.23	64.73	140	135.16	102.38	79.69
17h3898												
17h3899												
17h3900												
17h3901	52.24	53.43	360.85	362.96	5.42	4.99	65.26	64.56	190.74	238.62	14.75	12.9
17h3902	60.86	61.29	110.55	73.97	51.39	49.96	68.46	68.14	142.47	100.73	29.83	25.68
17h3903	97.61	96.98	185.43	159.03	69.47	57.3	98.69	98.71	110.17	103.8	61.23	41.16
17h3904	89.84	90.32	339.11	193.26	68.04	59.26	90.94	91	129.33	91.98	112.59	74.67
17h3905	0.07	0.2	84.31	201.31	90.13	30.13	0.07	0.18	78.38	112.14	30.8	20.96
17h3906	0.01	0.01					0.01	0.01	81.55	144.29	34.84	46.13
17h3907	0.31	0.33	84.23	103.82	79.78	63.95	0.02	0.02	64.44	121.27	33.36	61.8
17h3910	81.9	82.08	187.65	149.23	50.76	50.94	99.55	99.51	120.83	130.68	18.5	15.5
17h3913	8.34	8.52	480.9	432.53	11.64	11.23	12.33	10.77	717.56	665.78	2.28	3.12
17h3914	14.29	13.24	312.17	253.98	8.41	9.16	11.79	11.67	488.93	387.67	5.21	6.35
Mean	42.484545	42.580909					46.486364	46.3				
SD	38.711397	38.803999					41.84659	41.951824				
F-Test	0.9941203						0.9938192					

The three patients without data above had no PNH clones identified (events below LLOD were excluded).
The F-Test shows no significant statistical difference between the eBiosceince and BD CD157 PE reagent for either monocyte or granulocyte clones.

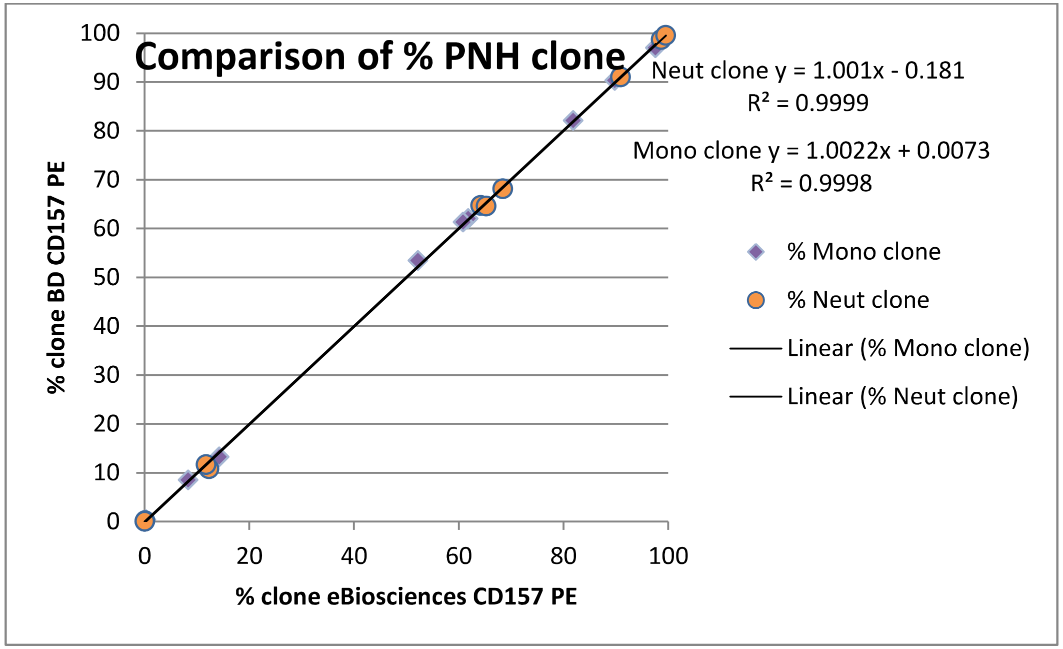

Regression analysis shows good correlation between clone sizes for both monocyte or granulocyte clones with R^2 values >0.99.

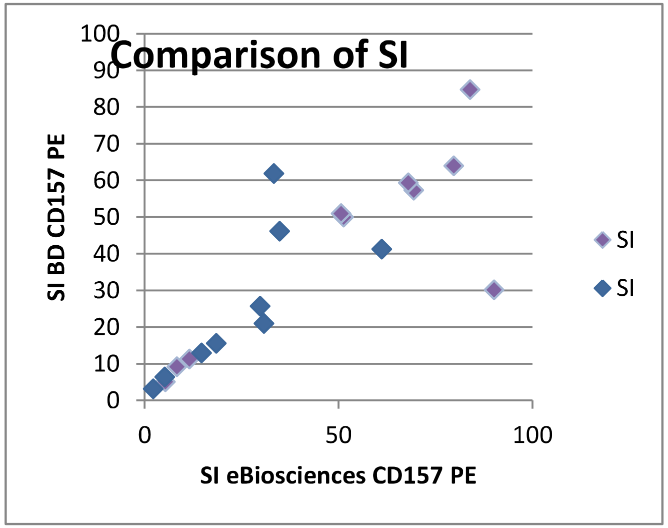

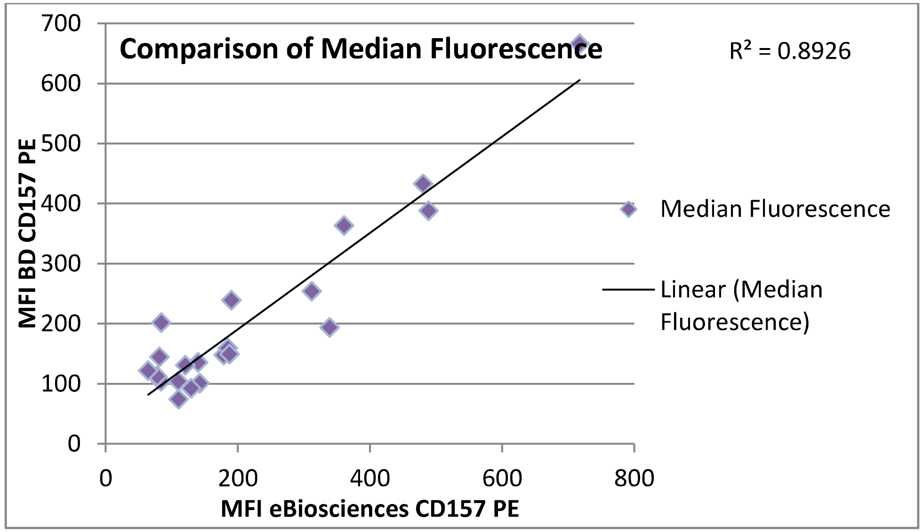

Acceptable SI's and MFI's were also observed. Individual patient data is supplied as an attachment to the Q-Pulse file (example below – file 1=eBio, file 2=BD) but analysis shows good distinction and separation of PNH and non-PNH clones.

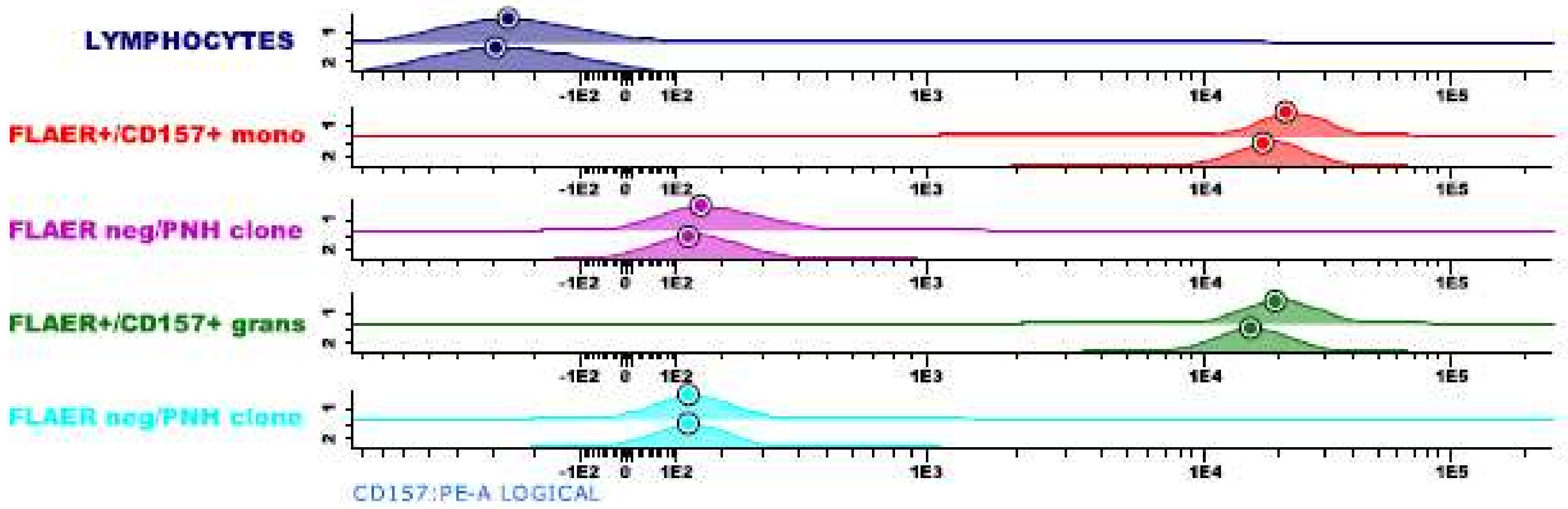

As seen in all previous NEQAS sample (using eBioscience CD157 PE), separation (SI) is reduced, likely due to the way the NEQAS samples are stabilised before distribution. This observation was also see with the BD CD157 PE however the SI values were slightly higher (see last two patients of the table).

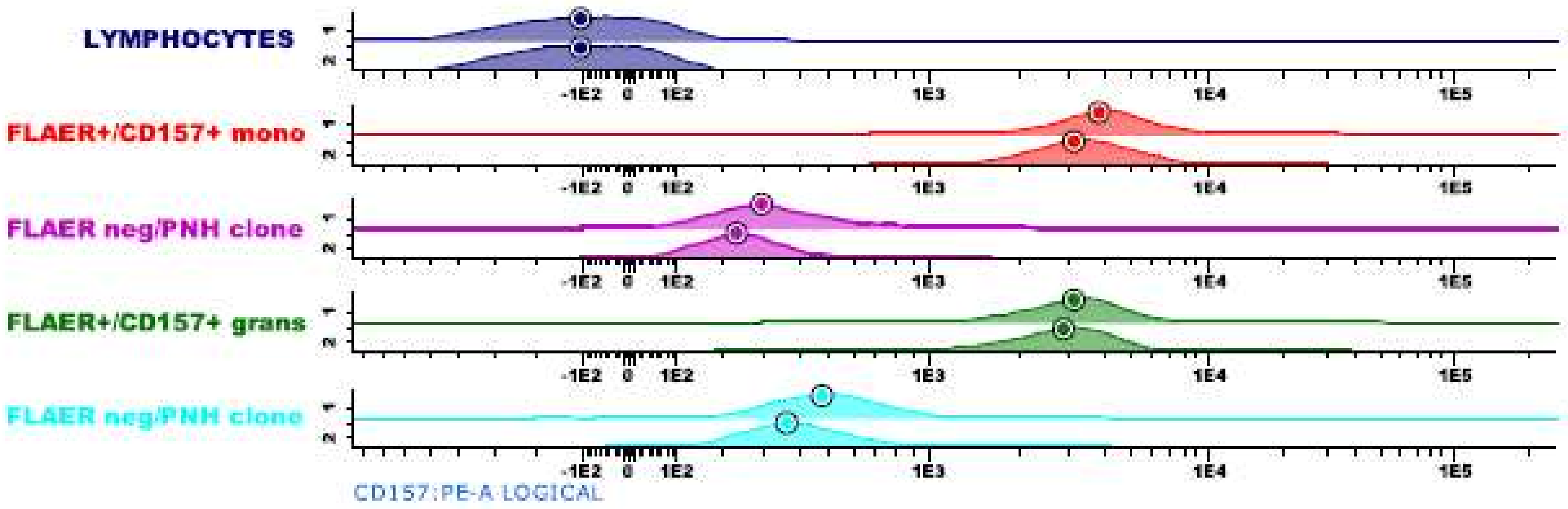

6. Review of Results against Acceptance Criteria

No	Revalidation Criteria<br>*As listed in Validation Plan*	Acceptance Criteria<br>*As listed in Revalidation Plan*	Acceptable/Not Acceptable<br>*(comments where applicable)*	Sign and date
RV1	EQA review	• No significant difference with reported value	Acceptable	KSanchez 5/9/2017
		• No significant difference with consensus results	Acceptable	KSanchez 5/9/2017
RV2	F-Test	• No significant difference between results (>0.98)	Acceptable	KSanchez 5/9/2017
RV3	Regression analysis	• Good correlation between data point (R^2 >0.95)	Acceptable	KSanchez 5/9/2017
RV4	Linearity	• Dilution assay not appropriate, linearity observed in regression analysis	Acceptable	KSanchez 5/9/2017

Revalidation completed satisfactorily: YES

Revalidation Study Manager Signature: …KSanchez .... Date: 05/09/2017

7. PROBLEM REPORT

Details of Problem(s)

N/A

Documentation Attached YES / NO No. of Pages

Person(s) Responsible

Actions Taken:

N/A

Performed By:

The problem(s) detailed above has / has not been fully resolved and further action is / is not required.

Revalidation Study Manager Signature: Date:

(**Documentation Retained for future reference by operational department**)

8. DOCUMENTATION REVIEW

The following documentation has been reviewed and amended appropriately, as a result of revalidation of the assay/process.
All additional documentation required has been prepared & issued and is listed below.

**Document Type & Identifiers**	**Comment** **(e.g. reviewed/ amended/ withdrawn/ implemented)**
N/A	

The above documentation has been reviewed / amended / introduced as appropriate

Revalidation Manager Signature: **Date:**

9. REVALIDATION REPORT SUMMARY & RECOMMENDATIONS

Report Recommendations

This validation report confirms the correlation of BD CD157 PE (clone SY11B5) with the existing (validated) eBioscience BD CD157 PE (clone SY11B5) (distributed by The Binding Site). No significant differences in clone sizes were seen, all clinical interpretation remained constant. This antibody can be put into immediate use following the withdrawal of the distribution by the Binding Site and use of any remaining stock. Stability data to confirm use in a cocktail to follow (see appendix 1).

Validation Manager Signature: **Date: 05/09/2017**

KSanchez

10. REVALIDATION REPORT AUTHORISATION

The staff below, as designated "Responsible Persons" approve and authorise the Revalidation Report.

Name	Position	Signature	Date
Alan Dunlop	Head of Immunophenotyping	Alan Dunlop	6/9/17

Comments where applicable:

11. BIBLIOGRAPHY

1. SOPs

1.1 LP-HAE-IM1035 Flow Cytometric Testing for PNH

2. Literature References

2.1 Sutherland *et al* (2014). 'Use of CD157 in FLAER-Based Assays for High-Sensitivity PNH Granulocyte and PNH Monocyte Detection.' *Cytometry Part B* **86B**:44-55

3. Policy References

3.1 N/A

4 Other Documents

4.1 N/A

12. APPENDIX 1

Cocktail stability containing PE CD157 PE

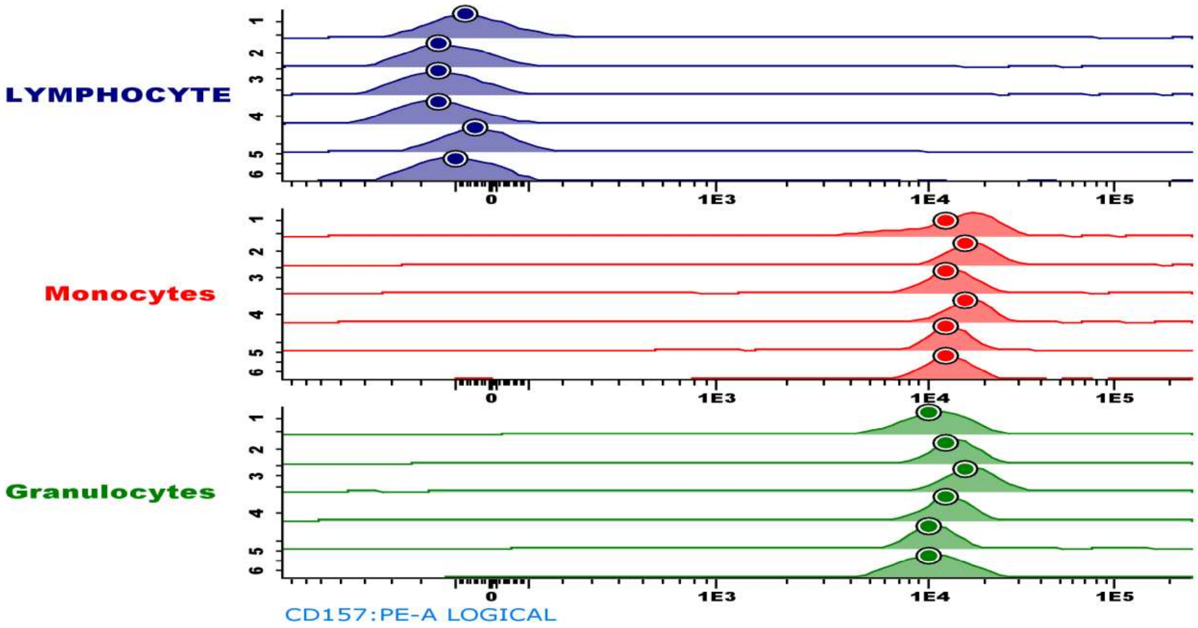

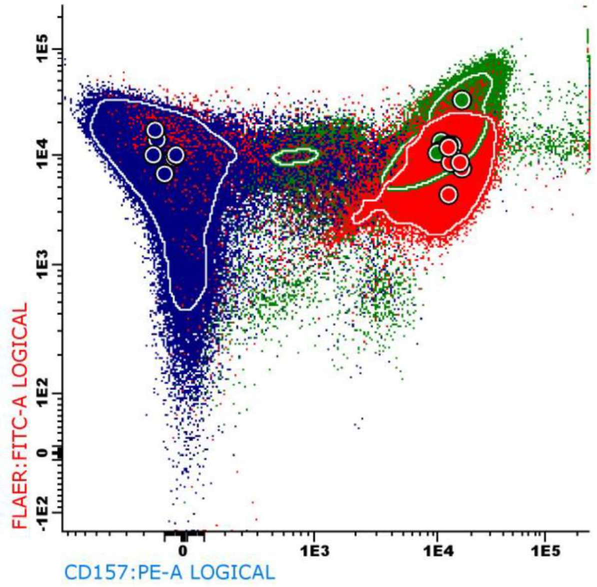

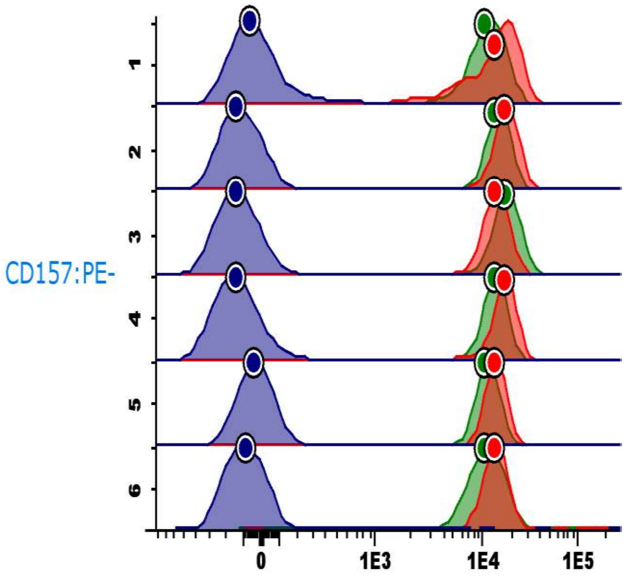

Antibody cocktails containing the new BD CD157 PE were made and tested over a period of 2 working weeks to ensure no changes in antigen expression were detected over time. In the population histogram above and the parameter histogram, below-right, the median fluorescence remained constant for both the negative control (lymphocyte) population (blue) and the CD157 positive monocyte (red) and granulocyte (green) populations. The data was collected on day 1 (file 1 on y axis), day 2 (file 2), day 4 (file 3), day 7 (file 4), day 9 (file 5) and day 11 (file 6) to cover 2 working weeks usage. When viewed overlaid in a dot plot (below-left), the median fluorescence of each time point (seen as a coloured dot) remains within the 2SD perimeter marker for each population type. It is therefore concluded that the WBC cocktail stability containing the new BD CD157 PE is stable for up to 11 days (2 working weeks).

Katy Sanchez 18/9/17
