# Supplementary material for: Clinical and prognostic significance of small paroxysmal nocturnal hemoglobinuria clones in myelodysplastic syndrome and aplastic anemia
Source: Leukemia. 2021 Mar 4;35(11):3223–31. doi: 10.1038/s41375-021-01190-9 (PMC8550969; doi:10.1038/s41375-021-01190-9)
Supplement: Supplementary file 4 — Supplementary material for flow cytometric testing [file 41375_2021_1190_MOESM4_ESM.pdf]

Flow Cytometric Testing for Paroxysmal Nocturnal Haemoglobinuria (PNH)

Version number	8
Author	Katy Sanchez
Authorised by	Charles Stanley manogaran
Issued on	08/07/20

Contents

1. Introduction ... 3
1.1 Purpose ... 3
1.2 Principle ... 4
1.3 Performance characteristics ... 4
2. Specimen Collection, Preparation and Storage ... 5
2.1 Patient preparation ... 5
2.2 Type of sample, container and additive ... 5
2.3 Specimen acceptance and rejection ... 5
2.4 Sample Preparation ... 6
3. Reagents, Materials and Equipment ... 6
3.1 Equipment and Materials ... 6
3.2 Reagents ... 6
4. Working Solutions and Antibody Cocktails ... 7
4.1 FACS Lyse Working solution: ... 7
4.2 WBC PNH Assay Cocktail ... 7
4.3 RBC PNH Assay Cocktail ... 8
5. Health and Safety precautions ... 9
5.1 COSHH ... 9
5.2 Risk Assessments ... 9
6. Calibration procedures ... 10
6.1 Metrological traceability and Measurement of Uncertainty ... 10
7. Quality control procedures ... 10
7.1 Internal QC ... 10
7.2 External QC ... 10
8. Methods ... 11
8.1 WBC PNH Assay ... 11
8.2 WBC PNH Gating/Analysis ... 12
8.3 WBC PNH Assay Acceptance and Interpretation ... 13
8.4 WBC PNH Manual Lysis Method (low cells acquired) ... 13
8.5 RBC PNH Assay ... 14
8.6 RBC PNH Gating/Analysis ... 15
8.7 RBC PNH Assay Acceptance and Interpretation ... 16
9. Requesting and Reporting of results ... 16
9.1 Requesting a PNH Test ... 16
9.2 Interpretation/Reporting/Authorisation of PNH Results ... 17
9.1 Printing and Monitoring of Reports ... 18
9.2 Amended reports ... 18
10. Limitations of the assay ... 18
11. References ... 19

1. Introduction

1.1 Purpose

Paroxysmal Nocturnal Haemoglobinuria (PNH) is a rare acquired genetic haematopoietic stem cell disorder with an annual rate of 1-2 cases per million. The prognosis without disease-modifying treatment is 10–20 years. Many cases develop in people who have previously been diagnosed with aplastic anemia or myelodysplastic syndrome (MDS). The fact that PNH develops in MDS also explains why there appears to be a higher rate of leukaemia in PNH, as MDS can sometimes transform into leukaemia.

In 2007, the drug 'Eculizumab' was approved for the treatment of PNH. It improves quality of life and decreases the need for blood transfusions. If a patient is treated with the anti-complement drug Eculizumab, the affected cells are effectively protected from the action of complement and percentages found in the blood will reflect the size of the PNH clone. Many PNH patients are treated or referred to Kings and it is common to find patients with PNH clone sizes in excess of 90%.

The classical presentation is one of a patient who passes red urine (haemoglobinuria) first thing in the morning, bone marrow failure (Aplastic Anaemia) and often with thrombosis or embolism in unusual sites i.e. the abdomen, liver, or cerebrum. The defect is due to the partial (Type II) or total (Type III) absence, or structural changes in the Glycosylphosphatidylinositol (GPI) anchor.

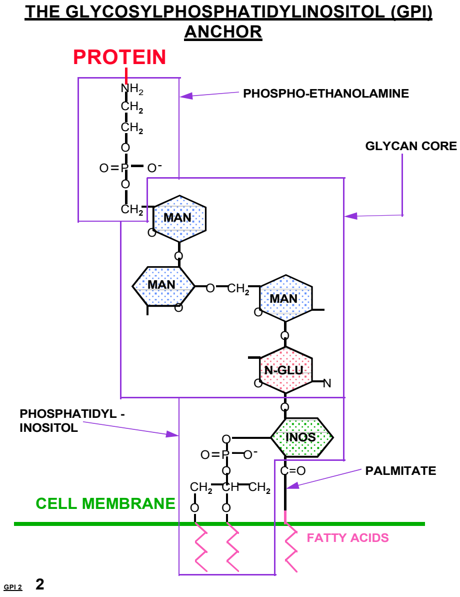

This anchor is attached one end into the cell membrane and the other end joins on to a variety of proteins. Deletions, point mutations, splice site mutations, insertion mutations and nonsense mutations in the gene, can all lead to different protein structures and conformations. The gene coding for this protein is the PIG-A gene (Phosphatidyl-inositol-glycan-Class A) which is a housekeeping gene located on the short arm of the X chromosome (Xp22.1). Absence / reduction / mutation of the GPI protein means that GPI linked proteins are either totally unable to attach, partially attach or attach in reduced numbers to the surface of all types of blood cells. In PNH, a vast majority of mutations have extremely severe functional consequences, (i.e., production of truncated proteins, leading to a complete GPI deficiency – the PNH type III phenotype), whereas missense mutations

are rare (usually resulting in the PNH type II phenotype). A number of observations support the idea that the PIG-A mutation itself is necessary, but not sufficient to cause PNH. In fact, the expansion of PNH cells over normal haematopoiesis remains a key step to develop the disease phenotype.

Examples of GPI linked molecules include Fcγ RIII (CD16) receptors, mediators of cell adhesion such as CD66b, CD14 on monocytes, some blood group antigens and most significantly from the haemolysis point of view, several complement defence proteins such as Decay Accelerating Factor (DAF- CD55) and the membrane inhibitor of reactive lysis (MIRL- CD59).

These proteins protect cells from the effects of autologous complement activation, which is thought to peak during the night hence the 'nocturnal' haemoglobinuria in PNH. The laboratory is able to use monoclonal antibodies and flow cytometry to check for the presence or absence of some of these GPI linked proteins on red and white blood cells and identify PNH clones.

1.2 Principle

Guidelines for the diagnosis and monitoring of PNH by flow cytometry were recently published (2010) by the International Clinical Cytometry Society (ICCS) and further recommendations were made in a paper by Sutherland et al 2012 and 2014 which have been adopted (and validated) within the laboratory. Both a red cell and white cell assays are used for diagnosis and disease monitoring.

Recommendations for the red cell assay were taken for the studies by Sutherland et al (2012). Of the CD59 conjugates tested in this paper, the MEM43 and OV9A2 clones conjugated to PE used with a CD235a FITC exhibited the best staining characteristics (signal-to-noise ratio) and allowed optimal separation between Type III and Type II PNH cells as well as separation from Type I (normal) cells while promoting minimal RBC agglutination. This study utilized the "racking" technique to disperse any aggregates immediately prior to data acquisition as part of the sample preparation regimen.

With respect to WBCs, a fluorescent labelled variant of the protein Aerolysin (FLAER) has been produced which binds selectively and specifically to the GPI anchor. This product has become the single most important part of the PNH assay due to the fact that it shows no lineage or maturation specificity, it is only specific for the GPI anchor i.e. if a cell is lacking the anchor, FLAER cannot bind and there will be no signal. A cocktail based on the selection of antibodies suggested by Sutherland et al (2014) that contains FLAER, CD157 (granulocytes), CD15 (granulocyte), CD64 (monocytes) and CD45 (pan-WBC) is used and validated within the laboratory for the assessment on granulocyte and monocyte clones.

1.3 Performance characteristics

RBC and WBC assays validated.
See associated documents on Q-Pulse QR-HAE-IM-VP0149, QR-HAE-IM-VR0149, QR-HAE-IM-VP0150, and QR-HAE-IM-VR0150.

2. Specimen Collection, Preparation and Storage

2.1 Patient preparation

No patient preparation required (e.g. fasting) or specified time of day for sample collection. Previous publications have noted circadian rhythms of RBCs and WBCs in circulation, however the day to night fluctuations are not significant enough to effect testing. Sampling is generally performed during the day (normal opening hours) when the circadian rhythm is stable. As a control measure, WBCs (or RBCs in PNH) are isolated as appropriate as part of the assay preparation and WBC (or RBC) have a minimum count applied during analysis on the flow cytometer.

Venesection should be performed by an appropriately trained professional to ensure good sample quality. See QPulse for Viapath Phlebotomy procedures and records. Patient and general blood test information can be found on the phlebotomy page of the Viapath website http://www.viapath.co.uk/departments-and-laboratories/phlebotomy-department

2.2 Type of sample, container and additive

2.2.1 EDTA anticoagulated peripheral blood samples are required.

2.2.2 Sample should be no more than 4 days old from point of venepuncture to analysis set up (internally validated see QR-HAE-IM-VR0177).

2.2.3 1ml minimum required. Samples less than the stated volume are processed where volume permits. Samples are visually checked for volume and clots on receipt/prior to processing. Comments are made as appropriate.

2.3 Specimen acceptance and rejection

Specimens are received via the HMDC reception where they have their initial quality controls checks made regarding sample labelling, patient ID and request cards. These specimens are recorded on (booked in) the LIMS (WinPath) and HMDC systems. Accepted samples will then be passed on to the laboratory (or other suitable sample pathway) and sample rejections are recorded on LIMS. Suitable assessments are made to accept/reject specimens as follows:-

2.3.1 Samples should be labelled with forename, surname and date of birth as a minimum requirement. Preferable they should also have a unique identification number (e.g. hospital number).

2.3.2 Samples should be accompanied by an appropriately completed request form with matching ID. Any discrepancies (or inadequacies) should be discussed with a senior BMS/laboratory manager and communication made with the referring clinician before deciding whether sample acceptance/rejection is required.

2.3.3 Samples should be received within 24 hours of being taken to allow testing to occur within the recommended timeframe of less than 4 days. Original recommendations are taken from 'Guidelines on the use of multicolour flow cytometry in the diagnosis of haematological neoplasms.' Johansson et al. BJH, 2014,165, 455-488 and later internally validated for extension to 4 days (QR-HAE-IM-VR0177). Times are recorded on LIMS.

2.3.4 Clotted samples are not appropriate for immunophenotyping.

2.4 Sample Preparation

2.4.1 Samples for PNH analysis are kept in a rack on the bench (room temperature) in HMDC reception to await collection by a member of the immunophenotyping personnel for a batch to commence. Any samples unprocessed at the end of the day should be moved into the sample fridge to be stored at 4°C (2-8°C) overnight.

3. Reagents, Materials and Equipment

3.1 Equipment and Materials

3.1.1 Flow Cytometer (BD FACS Canto): Work bench in Immunophenotyping lab.

3.1.2 BD Lyse Wash Assistance (LWA): Bench in HMDC reception/lab workspace

3.1.3 12 x 75 mm BD FACS tubes: Drawer 4.

3.1.4 Adjustable pipettes: On main workbench.

3.1.5 Plastic pipette tips: On main workbench.

3.1.6 Opaque Plastic block-tube rack: On shelf, above workbench.

3.1.7 Vortex mixer: On main workbench

3.1.8 Timers: On shelf, above main workbench

3.1.9 Mini centrifuges (Eppendorf 5702): On main workbench.

3.1.10 Universal containers: (In use) kept in box ABOVE the sink and main supply in large box underneath Instrunor in HMDC reception.

3.1.11 Plain 12 x 75 mm tubes (for RBC dilution): Drawer 3

3.2 Reagents

3.2.1 Distilled water: Distilled water keg on floor under workbench. Fill from tap in BSL film section. 'Squeezy' bottle kept by sink.

3.2.2 Phosphate buffered saline (PBS) (Inverclyde Biological): Keg on shelf near small reagent fridge and wash basin in lab. Stock kept in lab, HMDC reception and stock room (near BSL). Supplied ready to use by manufacturer. Expiry date on box. But once opened, the new expiry date will be four months from the date of opening. In use PBS in squeezy bottles (labelled as in use PBS) are kept on working bench during core working hours.(Kept in reagent fridge during non-working hours and weekends).

3.2.3 FACS Lyse (Becton-Dickinson): On shelf above main workbench. This comes as an x10 concentrate and needs to be diluted to working strength just prior to using.

3.2.4 Monoclonal antibodies: Reagent Refrigerator.

PNH antibodies are made in cocktail. These cocktails are validated and tested for its stability(stability tested and validated for 2 weeks). Each new batch of cocktail should be batch accepted by state registered scientist before any tests. Once batch accepted, these cocktail tubes are kept in the blue rack (labelled “PNH”) in large reagent refrigerator.

Please refer cocktail making SOP for further reference.

<u>WBC Assay</u>	FLAER (Alexa 488) – Cat# FL2S, Pinewood Scientific Services
	CD157 PE – Clone SY11B5, eBioscience
	CD15 APC – Clone HI98, Cat# 551376, BD Biosciences
	CD64 BV421 – Clone 10.1, Cat# 562872, BD Biosciences
	CD45 V500 – Clone 2D1, Cat# 655873, BD Biosciences
<u>RBC Assay</u>	CD235a FITC – Clone KC16, Beckman Coulter
	CD59 PE – Clone OV9A2, eBioscience

4. Working Solutions and Antibody Cocktails

4.1 FACS Lyse Working solution: (For manual lyse if both LWAs not working)

4.1.1 Note the number of tubes requiring FACS lysing.

4.1.2 Pipette and mix the appropriate amounts of x10 concentrated FACS Lyse into a universal container and add the appropriate volume of distilled water.

No of tubes	Vol. of FACS Lyse	Vol. of Distilled water
2	1.0ml	9.0ml
3	1.0ml	9.0ml
4	1.5ml	13.5ml
5	2.0ml	18.0ml
6	2.0ml	18.0ml
7	2.5ml	22.5ml
8	3.0ml	27.0ml

Shelf life - 7 days, once diluted at room temperature or 1 month if kept at 4°C. Write the date made up on the universal container.

4.2 WBC PNH Assay Cocktail

4.2.1 The stability WBC PNH cocktail has been validated up to 2 weeks.

4.2.2 According to expected workload cocktails should be pre-made, generally once a week. This will normally be 40 tests, but additional tubes can added later if necessary. Below is a table showing the cocktails

volumes for single tubes, 40 tests and various other volumes that may be made.

Reagent	Single Test	10 Tests	20 tests	40 Tests
**FLAER**	5µl	55µl	110µl	215µl
**CD157 PE**	5µl	55µl	110µl	215µl
**CD15 APC**	5µl	55µl	110µl	215µl
**CD64 BV421**	5µl	55µl	110µl	215µl
**CD45 V500**	5µl	55µl	110µl	215µl
Cocktail volume	**25µl**	**275µl**	**550µl**	**1075µl**

Note: volume for 10 tests is actually 10 plus 1 to allow for 10 'whole' volumes. The plus one allows for pipette tip coating or small inaccuracies which will slightly decrease the overall volume.

4.2.3 Create 25µl aliquots of the cocktail in 12x75mm BD FACS Tubes, cap, label and store in an opaque tube rack in the laboratory 'In-Use' fridge.

4.2.4 Cocktails should be given a batch number and recorded on the batch/cocktail worksheets in the laboratory.

4.2.5 A normal FBC (i.e. completed/waste sample from BSL, e.g. from AE) or a previously confirmed PNH negative sample (all antigens positive) should be used to test the cocktail and recorded on the cocktail/batch worksheet. If obtaining a normal FBC for control, this sample should have RBC and WBC indices within the reference ranges set by the haematology department and not known to be a Haem/Onc patient or on any treatments that may affect antigen expression. The sample should be less than 24 hours old.

4.3 RBC PNH Assay Cocktail

4.3.1 Prepare cocktails according to expected workload. Cocktail stability has been validated up to 2 weeks

Reagent	Single Test	10 Tests	20 tests	40 Tests
**CD235a FITC**	1.5µl	16.5µl	33µl	66µl
**CD59 PE**	0.5µl	5.5µl	11µl	22µl
**PBS**	18µl	198µl	396µl	792µl
Cocktail volume	**20µl**	**220µl**	**440µl**	**880µl**

Note: volume for 10 tests is actually 10 plus 1 to allow for 10 'whole' volumes. The plus one allows for pipette tip coating or small inaccuracies which will slightly decrease the overall volume.

4.3.2 Create 20µl aliquots of the cocktail in 12x75mm BD FACS Tubes, cap, label and store in an opaque tube rack in the laboratory 'In-Use' fridge.

4.3.3 Cocktails should be given a batch number and recorded on the batch/cocktail worksheets in the laboratory.

4.3.4 A normal FBC (i.e. completed/waste sample from BSL, from AE) or a previously confirmed PNH negative (all antigens positive) sample should be used to test the cocktail and recorded on the cocktail/batch worksheet. If obtaining a normal FBC for control, this sample should have RBC and WBC

indices within the reference ranges set by the haematology department and not known to be a Haem/Onc patients or on any treatments that may affect antigen expression. The sample should be less than 24 hours old.

5. Health and Safety precautions

5.1 COSHH

5.1.1 Sodium Azide (0.1%) – [HC-HAE-IM026]

5.1.2 FACS Lysing Solution – [HC-HAE-IM008]

5.1.3 Phosphate Buffered Saline – [HC-HAE-IM026]

5.2 Risk Assessments

This procedure has been given a **MEDIUM** risk assessment providing the precautions are adhered to due to the involvement of biological material. Refer to Risk Assessment: [HR-HAE-IM1035RA]

The following hazardous substances are used in this procedure

Substance name	Hazard	Precautions
Human Blood	B	Wear gloves. Avoid sharps injuries. Wear eye protection.
Sodium Azide (0.1%)	C I	Avoid skin contact and inhalation. Wear PPE.
FACS Lysing Solution	C I	Avoid skin contact and inhalation. Wear PPE.
Phosphate Buffered Saline	C I	Avoid skin contact and inhalation. Wear PPE.

IF IN ANY DOUBT CONSULT A SENIOR MEMBER OF STAFF

Key

F = Flammable. Avoid sparks / naked flames.
I = Hazardous by inhalation. Wear mask and handle substance within a safety cabinet.
C = Hazardous by skin contact. Wear protective gloves.
R = Radiation hazard. See specific instructions page in Laboratory Safety Manual in addition to COSHH reference.

B = Biohazard / Risk of Infection. Wear gloves and protective clothing.

In all circumstances;

- Wear protective clothing (laboratory coat, correctly and completely fastened and disposable gloves) at all times when handling biological materials.
- Wash hands thoroughly after handling any substances or after contact with biological materials.
- No smoking, drinking, eating or application of make up or contact lenses in designated laboratory areas.

6. Calibration procedures

Prior to running and analysing any sample for flow cytometry, the BD FACS Canto flow cytometers must have their daily start up and standardization performed. Refer to SOP LP-HAE-IM1030 - Flow Cytometer Start Up Procedure. Monthly calibration set up should be performed in compliance with SOP LP-HAE-IM1117 - BD FacsCanto II instrument setup and compensation.

6.1 Metrological traceability and Measurement of Uncertainty

No traceable standard available for flow cytometry or morphological assessments. See 'Qualitative Assessment for Measurement of Uncertainty – Immunophenotyping' for control measures [QF-HAE-IM002].

7. Quality control procedures

7.1 Internal QC

Details on samples/slides and request forms should be matched. Any unmatched sample must be rejected or held back and the requesting clinician informed of any discrepancies.

See 'Qualitative Assessment for Measurement of Uncertainty – Immunophenotyping' for control measures [QF-HAE-IM002].

There is no commercial PNH control available, however, due to the heterogeneous nature of the PNH defect, unless the patient is on Eculizumab (patients on Eculizumab can reach clone sizes of 100%), there will always be a population of residual normal cells that will bind the antibody to the corresponding antigen providing evidence that the antibodies in use are viable. These are internal assay population controls. Samples are always run in batches where a varied amount of normal, type II and type III PNH clones are likely to be detected. See cocktail working solutions for control of batches.

7.2 External QC

External QC samples for PNH analysis are provided by 'UK NEQAS for Leucocyte Immunophenotyping' which is an international External Quality Assessment (EQA)/Proficiency Testing (PT) provider hosted by, and is legally accountable to, Sheffield Teaching Hospitals NHS Foundation Trust.

NEQAS samples are managed in the same way as patient samples. NEQAS samples should be stored as described on the accompanying form (normally 2-8°C) until the final NEQAS 'satisfactory' report is received. This allows availability of sample should any investigation for poor performance be required.

8. Methods

PNH analysis involves two assays for accurate diagnosis; a WBC assay (which assesses the presence of GPI linked proteins on both monocytes and granulocytes) and a RBC assay.

The assays can be run independently during the day and because the WBC assay tubes can be placed on the Lyse Wash Assistant (LWA), these tubes can be batched with other non-PNH leukaemia/lymphoma work running on the LWA. Results from both assays are required for a final report.

NOTE: Use a fresh pipette tip for each tube and each antibody to minimise contamination, always add the antibody/blood to the bottom of the tube to ensure accuracy of volumes/mixing/lysing/staining.

8.1 WBC PNH Assay

8.1.1 Remove enough WBC PNH cocktail tubes from the reagent fridge, one tube per sample in the batch (or create as per previous instruction).

8.1.2 Label the tubes with the patients' name and laboratory accession number or with WinPath barcode/ID stickers. Initial the "assay set up" section of the worklist. A second check must be performed to ensure correct blood into correctly labelled tube. This is to check that the samples are arranged according to the worklist and secondary tubes correspond to primary tubes. This second check must be recorded on the worklist as a second set of initials in the "assay set up" column.

8.1.3 Pipette 100µl of well mixed peripheral blood into the correspondingly labelled WBC PNH cocktail tube. Wipe the tip before expulsion of the blood to avoid transferring blood onto the side of the tube.

8.1.4 Mix using the vortex mixer and incubate in the dark, at room temperature for 15 minutes (maximum one hour).

8.1.5 Load the carousel onto the LWA, press the "FACS Lyse" button and close the lid to start the protocol. If the lid is closed before pressing the "FACS Lyse" button, then the green RUN button will need to be pressed to start the protocol. (Take note on the processing time to return to collect the samples).

8.1.6 At the end of lysis and washing, open the lid on the LWA, remove the carousel. Close the LWA lid.

8.1.7 Take the carousel to the BD FacsCanto II on which it is to be run and ensure the worklist has been imported from workflow manager (WFM). (See [LP-HAE-IM1113] Use of the BD Workflow Manager). Transfer all the tubes from carousel into the blue SARSTEDT rack according to the number order listed in worklist. Import worklist into FACS mode and verify the sample numbers against the worklist to ensure the samples are in the correct order according to the worklist. Initial the "loaded/ID checks" section of the worklist. These checks must be repeated by a second checker, which should then be recorded on the "loaded/ID checks" section of the worklist as a second set of initials.

8.1.8 Ensure all assays to be run on the manual mode are WBC samples and therefore can use the standard EuroFlow settings. Additionally ensure the experiment has been linked to the EuroFlow settings and all settings are correct.

8.1.9 Start running the samples manually and acquire at least 500,000 events in total and ensure at least 100,000 combined events in P2 & P3 (granulocyte) gates.

8.1.10 Once the batch has completed running on the flow cytometer, select the batch by clicking on the label on the tree on the left on the (Diva) screen, right click to select 'Batch Analysis'.

8.1.11 Once 'Batch analysis' has started, the software will allow you to see each sample in the batch in turn. Ensure all the gates are placed correctly around the populations and adjust as necessary. Repeat this process for each sample in the batch until the end is reached. See 'WBC PNH gating/analysis' and 'WBC PNH assay acceptance' sections in this SOP.

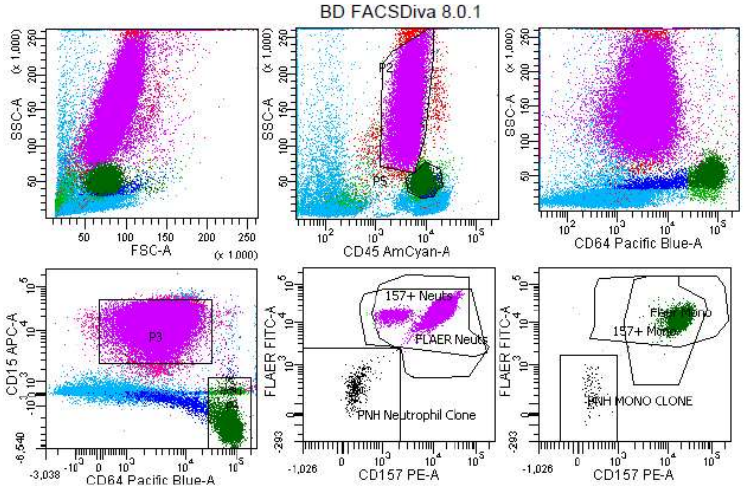

8.1.12 To enable results to be sent back to the workflow manager (and then to LIMS), <u>you must close the Diva software.</u> Results will not be sent to the WFM if the DIVA software is not completely closed, you should see a message pop up on the screen to indicate that the results were successfully completed.

8.1.13 Refresh the WFM to ensure results are successfully received. Results can then be verified from the Winpath queue.

8.2 WBC PNH Gating/Analysis

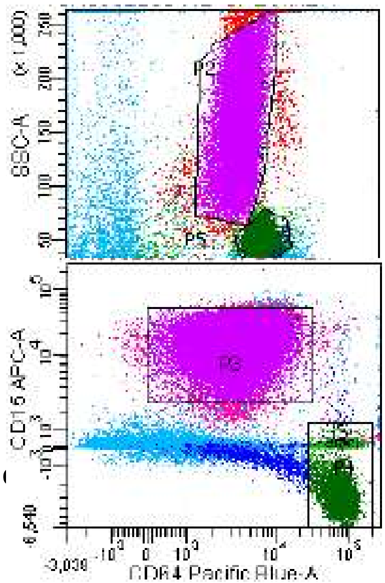

8.2.1 In the CD45 vs SS plot, ensure the P2 gate is tightly drawn around the granulocytes which express CD45 moderately and have medium to high side scatter. Also ensure that the P5 gate is placed tightly around the monocyte population which expresses CD45 more brightly and has lower side scatter.

8.2.2 In the CD64 vs CD15 gate, place the P3 gate around the neutrophil population (CD64 moderate,

CD15 bright) and the P4 gate around the monocytes (CD64 bright and CD15 negative).

8.2.3 The neutrophil populations gated in P2 and P3 are then used to populate the CD157 vs FLAER neutrophil plot. Ensure the '157+ Neuts' and 'FLAER Neuts' gates are around the positive populations. The negative population will be in the bottom-left of the dot plot, place the 'PNH Neutrophil Clone' gate around any cells that fall here.

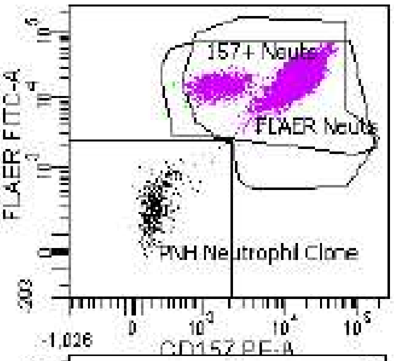

8.2.4 The monocyte populations gated in P4 and P5 are then used to populate the CD157 vs FLAER monocyte plot. Ensure the '157+ Mono' and 'FLAER Mono' gates are around the positive populations. The negative population will be in the bottom-left of the dot plot, place the 'PNH Mono Clone' gate around any cells that fall here.

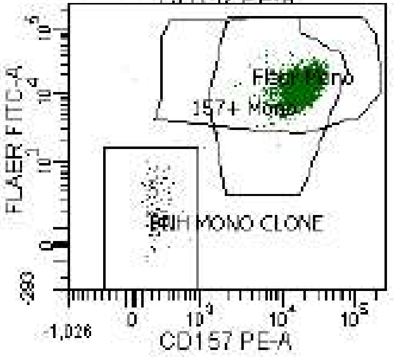

8.3 WBC PNH Assay Acceptance and Interpretation

8.3.1 Normal analysis will collect 100,000 cell events. Less than 50 'clone' events should be considered as the absence of PNH and beyond the lower limit of detection (LLOD).

8.3.2 More the 50 clone events should be considered as a true PNH clone.

8.3.3 A minimum of 50 'clone' events must be collected to be considered as presence of a PNH clone. If the amount collected is between 5 and 50 or the 100,000 minimum cell count was not reached (which can occur in patients with aplastic anaemia), the assay is considered invalid. In these cases discuss with senior scientists to decide whether samples should be repeated with multiple tubes in order to capture more events. For cases with increased un-lysed cells, please refer to section 8.4.

8.4 WBC PNH Manual Lysis Method (in the cases with increased un-lysed cells)

8.4.1 Remove a WBC PNH cocktail tube from the reagent fridge. Label this tube with the patients' name and laboratory accession number.

8.4.2 Pipette 300µl of well mixed PB into the WBC PNH cocktail (wipe the tip to avoid transferring blood onto the side of the tube).

8.4.3 Mix using the vortex mixer and incubate in the dark, at room temperature for 15 minutes (maximum one hour).

8.4.4 After incubation fill the tube with BD FACS lyse diluted to 1:10. Cap the tube and mix well by inversion. Lyse on the bench in an opaque block rack for 15 minutes.

8.4.5 Centrifuge the tube in the Minifuge for 5 minutes at 2800 rpm.

8.4.6 Remove the supernatant (lysate) using a plastic Pasteur pipette. DO NOT TIP OFF THE SUPERNATANT.

8.4.7 Mix and top up with PBS. Spin again at 2800rpm for 5 minutes.

8.4.8 Remove the supernatant using a plastic Pasteur pipette. DO NOT TIP OFF THE SUPERNATANT.

8.4.9 Re-suspend the cell pellet and add 350ul of PBS to the tube.

8.4.10 Process on the flow cytometer using the CD157 analysis template and follow the steps from section 8.1.7 onwards.

8.5 RBC PNH Assay

8.5.1 Remove enough RBC PNH cocktail tubes from the reagent fridge, one tube per sample in the batch (or create as per previous instruction).

8.5.2 Label the tubes with the patients' name and laboratory accession number or with WinPath barcode/ID stickers. Initial the "assay set up" section of the worklist. A second check must be performed to ensure correct blood into correctly labelled tube. This is to check that the samples are arranged according to the worklist and secondary tubes correspond to primary tubes. This second check must be recorded on the worklist as a second set of initials in the "assay set up" column.

8.5.3 Gently mix the sample by inversion and dilute peripheral blood by adding 5µl mixed blood to 495µl PBS in a labelled plain 12x75mm test tube.

8.5.4 Pipette 50µl of mixed diluted blood sample into the correspondingly labelled RBC cocktail aliquot tube and gently mix by 'up-and-down' pipetting.

8.5.5 Further mix by gently swirling the sample using a slow speed vortex, taking care not to generate aerosols.

8.5.6 Incubate in the dark for 20 min at room temperature (incubation times of up to 60 min generates identical results – Sutherland et al 2012).

8.5.7 Wash twice with PBS by centrifugation as is required to optimize separation of Type I, II, and III red blood cells (spin at 2800rpm for 2 minutes, remove supernatant with a Pasteur pipette).

8.5.8 Re-suspended in 0.5ml of PBS.

8.5.9 The sample should be "racked" (dragged vigorously across a hard plastic or metal test tube rack several times) to disrupt any RBC aggregates generated by the staining/washing procedure immediately before acquisition on the cytometer.

8.5.10 Samples should be acquired immediately, as delays longer than 15 min after the final washing step typically show decreased CD235a staining.

8.5.11 Import the worklist from workflow manager (WFM) into FACS mode. (See [LP-HAE-IM1113] Use of the BD Workflow Manager). Verify the sample numbers against the worklist to ensure the samples are in the correct order according to the worklist. Initial the "loaded/ID checks" section of the worklist. These checks must be repeated by a second checker, which should then be recorded on the "loaded/ID checks" section of the worklist as a second set of initials.

8.5.12 Ensure all assays to be run on manual mode are RBC samples and are using the current RBC settings (standard EuroFlow settings are for WBCs only).

8.5.13 Start running the samples manually and collect at least 100,000 events in P4 gate.

8.5.14 Once the batch has completed running on the flow cytometer, select the batch by clicking on the label on the tree on the left on the (Diva) screen, right click to select 'Batch Analysis'.

8.5.15 Once 'Batch analysis' has started, the software will allow you to see each sample in the batch in turn. Ensure all the gates are placed correctly around the populations and adjust as necessary. Repeat this process for each sample in the batch until the end is reached. See 'RBC PNH gating/Analysis' and 'RBC PNH Assay Acceptance' sections in this SOP.

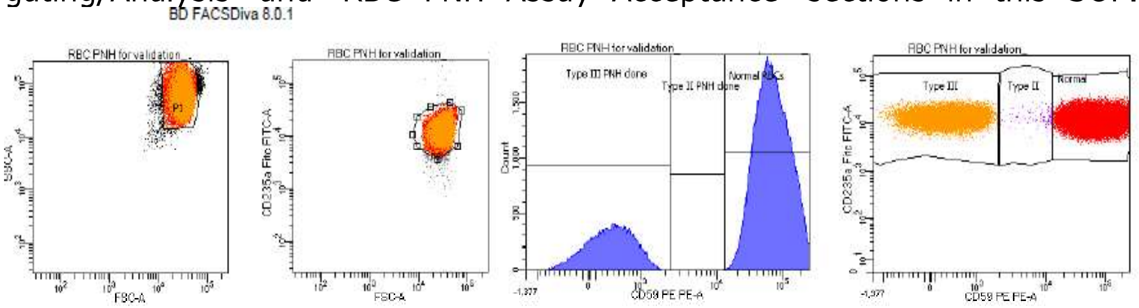

8.5.16 To enable results to be sent back to the workflow manager (and then to LIMS), <u>you must close the Diva software.</u> Results will not be sent to the WFM if the DIVA software is not completely closed, you should see a message pop up on the screen to indicate that the results were successfully completed.

8.5.17 Refresh the WFM to ensure results are successfully received. Results can then be verified from the Winpath queue.

8.6 RBC PNH Gating/Analysis

8.6.1 In the FS vs SS plot, ensure the P1 gate is placed tightly round the main RBC population. Leave events out of the gate that have too high forward scatter caused by doublets or clumps of erythrocytes.

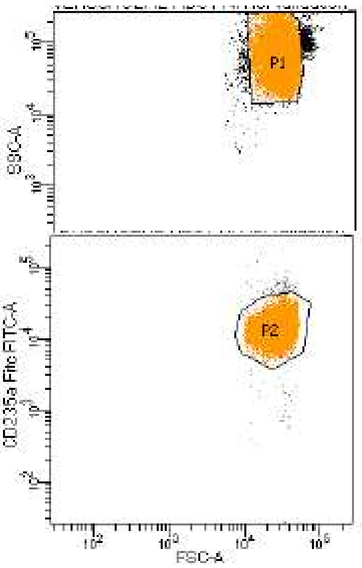

8.6.2 The cells from P1 are used to populate the FS vs CD235a plot, gate (P2) the RBCs leaving out cells gate that have too little CD235a expression or too much (the latter being caused by doublets or clumps of erythrocytes).

8.6.3 The cells from P2 are used to populate the CD59 histogram and dot plot. In the CD59 histogram, place the 'Normal RBCs' gate around the CD59 bright peak on the far right. The 'Type II PNH clone' gate should be placed around any peak detected to the left of the normal population and shows moderate CD59 expression. The 'Type III PNH clone' gate should be placed around any peak detected in the negative region of the histogram.

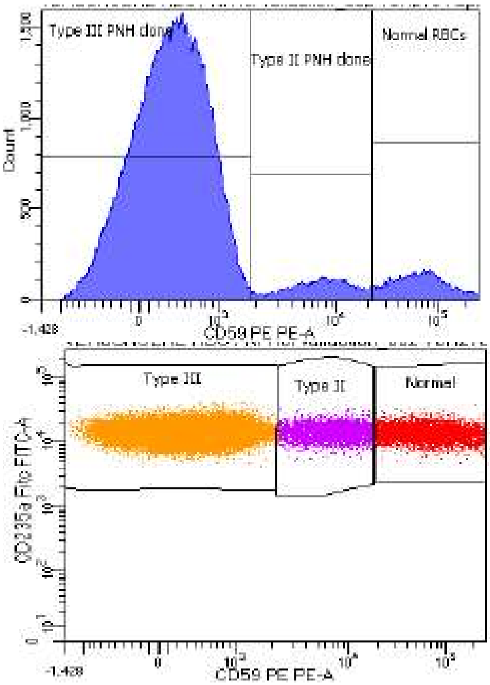

8.6.4 The same principle should be applied to the CD59 vs CD235a dot plot. Guidance on population edges/position may be taken from the histogram which also shows CD59 expression on the x axis.

8.7 RBC PNH Assay Acceptance and Interpretation

8.7.1 A minimum of 100,000 RBCs should be acquired. If two or more events are displayed in the Type III PNH RBC region data acquisition should be continued until 1million events are acquired. These results can be appended to the original data.

8.7.2 At least 50 'clone' events are required to describe a population as having PNH – This will give a sensitivity of detection down to 0.005% if 1 million events are counted. If less than 50 cells are counted in 1 million – report as "No evidence of a PNH Clone."

9. Requesting and Reporting of results

9.1 Requesting a PNH Test

9.1.1 Samples should be pre-requested on the LIMS and IOG system in the HMDC reception with the testcode 'PNH'. Instruction can be found in the [LP-HAE-IM1001] 'Winpath use' SOP.

9.1.2 This will trigger two tests to be generated on the workflow manager; PNHW (WBC PNH assay) and PNHR (RBC PNH assay).

9.2 Interpretation/Reporting/Authorisation of PNH Results

9.2.1 After batch analysis on the flow cytometer, numerical results will automatically transmit to LIMS. If a sample has had to be repeated manually or for any reason that the workflow manager fails to send a result, this must be inputted manually on to LIMS (Winpath). To do this, search for the laboratory accession number on Winpath, double click on the sample in the list. From there you can review by selecting 'Results' and input results by selecting 'Amend'.

9.2.2 Percentages from the Stats box for CD157+ and FLAER+ monocytes and granulocytes, CD59+ RBCs as well as the PNH clone sizes for granulocytes, monocytes, RBCs, Type II and Type III are required. This is an example of data entry on LIMS:

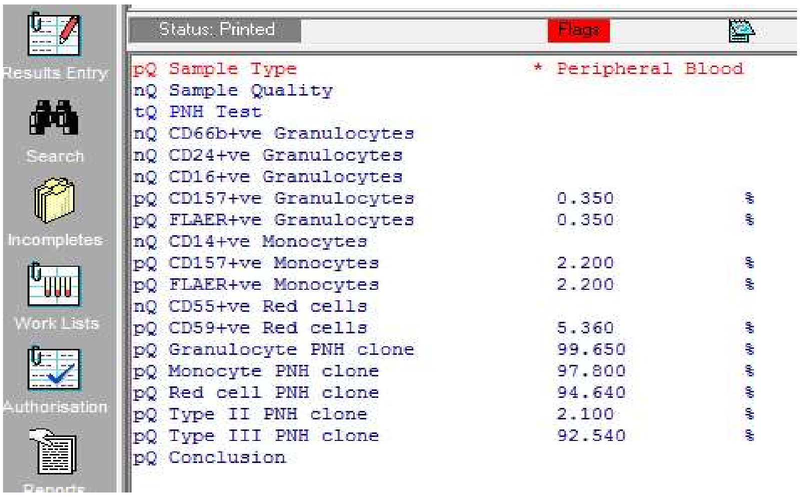

pQ Sample Type	* Peripheral Blood	
nQ Sample Quality		
tQ PNH Test		
nQ CD66b+ve Granulocytes		
nQ CD24+ve Granulocytes		
nQ CD16+ve Granulocytes		
pQ CD157+ve Granulocytes	0.350	%
pQ FLAER+ve Granulocytes	0.350	%
nQ CD14+ve Monocytes		
pQ CD157+ve Monocytes	2.200	%
pQ FLAER+ve Monocytes	2.200	%
nQ CD55+ve Red cells		
pQ CD59+ve Red cells	5.360	%
pQ Granulocyte PNH clone	99.650	%
pQ Monocyte PNH clone	97.800	%
pQ Red cell PNH clone	94.640	%
pQ Type II PNH clone	2.100	%
pQ Type III PNH clone	92.540	%
pQ Conclusion		

9.2.3 When you reach the conclusion field, there are several comments that can be entered. Pressing F7 twice will bring up a menu which can be filtered by selecting 'Immunophenotyping'.

- By typing '@PNHN' the comment 'No Immunophenotypic evidence of type II or type III PNH abnormality' text will be generated.
- When a PNH clone is detected, write the free text comment 'PNH clone detected in RBCs and WBCs' as appropriate.
- If the clone is very small (<1%) then free text the comment 'Small population of GPI deficient cells detected, ?clinical significance'.
- In some cases of autoimmune conditions, the RBC assay may show reduced CD59 expression but have no evidence of a granulocyte or monocyte PNH clone in the WBC assay. This should be reported as 'Reduced CD59 expression detected but in the absence of a WBC GPI deficient clone, this result is not consistent with PNH'.

9.2.4 When results are automatically transmitted via the Workflow Manager, the conclusions can be added at the result authorisation point.

9.2.5 Results can be authorised by a suitably competent HCPC registered scientist. If results were entered manually, they must be authorised by a second person.

9.2.6 To authorise a result in Winpath, select the 'Authorisation' tab and then select the 'Immunophenotyping' list. From the list you can select the samples you wish to authorise by double clicking on them. This will open the patient encounter. Add the appropriate interpretative comment as required, check the numerical results against the PDF saved on the drive

and when you are pleased the result is correct, select the 'Authorise' tab to release the result.

9.1 Printing and Monitoring of Reports

Reports are generally not generated for King's in-patients, but external patients will need to have a final report printed. Call up the relevant results and click the icon next to the flag (i.e. the magnifying glass over a page of text) to get a print preview. When the print preview appears; click the 'Print' icon in the top left of the page and select the appropriate printer (see LP-HAE-IM1007 for comprehensive instructions).

9.2 Amended reports

If an amended report is required, the original results screen should be called up (see SOP IH-1007); place the cursor on the line of the result that requires amending. Using Control O (for overwrite) will allow you to amend a result. Click the 'save' button at the bottom, a request will be made as to why the change has been made, this must be filled in with an appropriate comment to complete the audit trail. The result will automatically un-authorise and go back to the authorisation queue ready to be re-authorised.

10. Limitations of the assay

Because of the heterogeneous levels of all protein expression, care must be taken when assigning gates and cut-off points. Seek advice from a senior member of staff before reporting if you are not sure about placement of gates. Collect as many events as possible to ensure better accuracy, precision and sensitivity. The set minimum number of events can be difficult to achieve in aplastic patients or Hypoplastic MDS patients. A minimum of 50 'clone' events are needed to reliably define a population.

Monocytes are often very low in number and it is essential that at least 500 are collected to be reported on. If an obvious PNH clone has been identified with more than 50 neutrophils, but only 20 or so monocytes appear to show PNH, it is acceptable to report the monocyte PNH percentage recorded on the flow cytometer.

The PNH assay can be affected by a number of factors such as:

1. The age of the sample: Samples over 4 days old are not suitable for granulocyte / monocyte analysis of PNH and samples over 4 days old are not suitable for red cell analysis of PNH. It is recommended that samples that arrive late on a Friday (or a day prior to a bank holiday weekend) should at least have their granulocytes and monocytes screened, as these may become degraded over the weekend / holiday.

2. The presence of increased numbers of immature myeloid cells can complicate the interpretation of PNH analysis. Immature myeloid cells will have a lower or a negative expression of markers such as CD15, and CD157, possibly resulting in generating false "PNH positive" results.

3. Analysis of PNH should <u>not</u> be carried out on bone marrow samples for the above reason.

4. Increased numbers of nucleated red cells, reticulocytes, sickle cells or target cells can have a detrimental impact on PNH screening and

interpretation, including difficultly in lysing red cells and possibly lower expression of the red cell antibodies (CD59) resulting in false "PNH positive" results.

11. References

- Guidelines for the diagnosis and monitoring of paroxysmal nocturnal hemoglobinuria and related disorders by flow cytometry. Borowitz et al. Cytometry B Clin Cytom 2010 Jul;78(4):211-30.
- Practical guidelines for the high-sensitivity detection and monitoring of paroxysmal nocturnal hemoglobinuria clones by flow cytometry. Sutherland et al. Cytometry B Clin Cytom 2012 Jul;82(4):195-208.
- Use of CD157 in FLAER-Based Assays for High-Sensitivity PNH Granulocyte and PNH Monocyte Detection. Sutherland et al. Cytometry B Clin Cytom 86B:44–55 (2014)
